# Polyplex of peptide-mannan and RNA for intranasal delivery of TGF-β siRNA in treatment of pulmonary fibrosis

**DOI:** 10.1016/j.bioactmat.2026.02.006

**Published:** 2026-02-06

**Authors:** Bailin Feng, Abhalaxmi Singh, Yiqing Yang, Philana Phan, Han Xu, Yuli Zhu, Jennifer Huang, Vrushank Sastry, Zongmin Zhao, Ying S. Hu, Gang Cheng, Asrar B. Malik, Ying Liu

**Affiliations:** a Department of Chemical Engineering, University of Illinois Chicago, Illinois, 60607, United States; b Department of Pharmacology & Regenerative Medicine, University of Illinois Chicago, Illinois, 60607, United States; c Department of Pharmaceutical Sciences, University of Illinois Chicago, Illinois, 60612, United States; d Department of Chemistry, College of Liberal Arts and Sciences, University of Illinois Chicago, Chicago, IL, 60607, United States; e University of Illinois Cancer Center, Chicago, IL, 60612, United States; f Department of Biochemistry and Molecular Genetics, College of Medicine, University of Illinois Chicago, Chicago, IL, 60607, United States; g Department of Biomedical Engineering, Colleges of Engineering and Medicine, University of Illinois Chicago, Chicago, IL, 60607, United States; h School of Engineering, Westlake University, Hangzhou, 310024, China; i Cell Biologics, 2201 W Campbell Park Drive, Chicago, IL, 60612, United States

**Keywords:** Ionizable peptide, Macrophage, Polymeric nanoparticle, Lipid nanoparticle, Gene therapy

## Abstract

Pulmonary fibrosis is a progressive, severe respiratory disease, often considered terminal, with a typical life expectancy of only a few years. It is marked by excessive deposition of extracellular matrix proteins, driven by a complex interplay of profibrotic signaling pathways, including contributions from monocyte-derived alveolar macrophages (Mo-AMs) and various immune and stromal cells. In this study, we present a peptide-mannan conjugate nanoparticle (PMNP) platform for the targeted delivery of transforming growth factor-β small interfering RNA (TGF-β siRNA) aimed at halting and reversing pulmonary fibrosis. The nanoparticles of TGF-β siRNA and peptide-mannan conjugates, generated through a solvent-free and easily scalable process, were administered intranasally to specifically target the alveolar macrophage population. In fibrotic models, these nanoparticles effectively reduced Mo-AM infiltration, reprogrammed the macrophage phenotype, and significantly reduced collagen deposition. Our findings suggest that intranasal delivery of TGF-β siRNA via PMNP offers a promising, easily self-assembled, and patient-friendly therapeutic approach for the treatment of lung fibrosis.

## Highlights

• Peptide–mannan conjugate targets fibrotic alveolar macrophages via airway delivery.
• Solvent-free process yields stable RNA-loaded peptide–mannan nanoparticle (PMNP).
• TGF-β siRNA reprograms pro-fibrotic macrophages and reduce fibrosis.

## Graphical abstract

**Figure:**
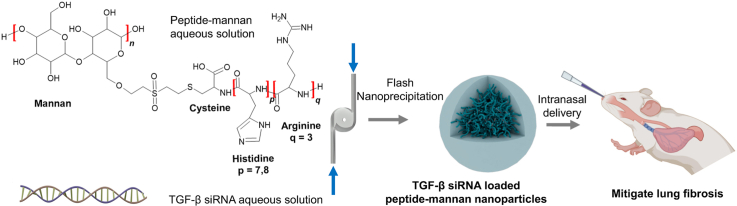

## 1 Introduction

The development of small interfering RNA (siRNA)-based therapies represents one of the most significant advances in modern disease treatment, offering the ability to selectively silence pathological mRNA transcripts. Once delivered into cells, siRNA molecules guide the RNA-induced silencing complex (RISC) to degrade the specific mRNA, thereby reducing the production of disease-associated proteins [1,2]. However, effective siRNA delivery demands a suitable carrier system, because unprotected siRNA is readily degraded and cannot efficiently cross cellular membranes. Such carriers must shield siRNA from premature degradation *in vivo*, overcome various biological and immune barriers, and facilitate endosomal escape to achieve successful gene silencing [[3], [4], [5]]. RNA delivery approaches are generally categorized into viral and non-viral systems. While viral vectors often offer high transfection efficiency, concerns regarding toxicity and safety remain significant drawbacks. Viral vectors cannot be effectively used in patients with pre-existing immunity to the viral capsid proteins. Over the last two decades, research on non-viral delivery systems has primarily focused on cationic polymers and lipids, phospholipids, and ionizable lipids [[4], [5], [6], [7], [8], [9]]. Lipid nanoparticles (LNPs) have demonstrated substantial success in nucleic acid delivery, as seen with the SARS-CoV-2 mRNA vaccines [10,11]. Following the U.S. Food and Drug Administration (FDA)'s approval of the first LNP-based siRNA therapeutic, Onpattro, for hereditary transthyretin amyloidosis (hATTR) [12], five more siRNA therapeutics have been approved by FDA, with several more in phase III clinical trials [2,[13], [14], [15]]. However, none of these additional FDA-approved siRNA therapeutics use LNP for their delivery. Moreover, all of these siRNA therapeutics require parenteral administration.

For the treatment of lung diseases, a targeted gene delivery system directed specifically to the lungs is highly desirable. LNPs, however, primarily accumulate in the liver, limiting LNP applicability for pulmonary indications. Although some studies have reported the design of LNP compositions that preferentially circulate to the lungs, the structure-function correlation is not clear for the optimization of such delivery vesicles [16,17]. Direct aerosol or intranasal [[18], [19], [20]] administration may be more efficient and patient-friendly than injectable routes, as it avoids the undesired accumulation of vesicles in non-target tissues and organs before reaching the lungs, thereby preserving lung-specific bioavailability and reducing potential side effects [21]. Moreover, this approach takes advantage of the lungs’ large surface area, providing direct access for delivering therapeutic agents to alveolar regions.

During lung fibrosis, alveolar macrophages (AMs) and monocyte-derived interstitial macrophages (IMs) accumulate in the lung and secrete profibrotic mediators that drive the activation of fibroblasts. The resulting myofibroblasts are the primary effector cells responsible for excessive collagen deposition, which obstructs alveolar pores, diminishes lung function, and can ultimately lead to respiratory failure. Among the key signaling molecules, transforming growth factor-β (TGF-β) is considered a potent inducer of fibrosis. It promotes the differentiation of fibroblasts into myofibroblasts, driving excessive extracellular matrix (ECM) deposition and the progressive decline in lung function. [22,23]. Therapeutic administration of TGF-β siRNA lowers TGF-β protein levels, thereby disrupting the signaling pathways that drive fibroblast activation and excessive collagen deposition in the lung. However, because TGF-β is involved in multiple immune-regulatory pathways, broad and sustained suppression may result in undesirable and unpredictable long-term effects, including tumorigenesis [24], impaired tissue repair [25], altered immune responses [26,27], and increased susceptibility to injury or infection [[28], [29], [30]]. To address these concerns, the strategy of this study is to achieve localized and transient TGF-β silencing through intranasal administration of siRNA, enabling a non-permanent, time-limited knockdown that mitigates fibrotic signaling while minimizing potential long-term adverse effects.

**Fig. 1:**
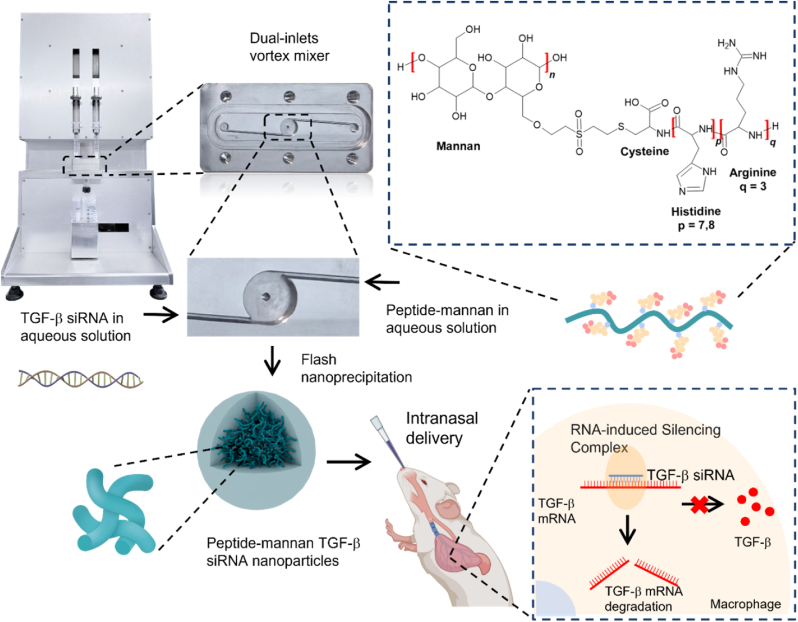
**Schematic illustration of peptide-mannan mediated TGF**-β **siRNA delivery**. The diagram depicts the formation of the peptide-mannan TGF-β siRNA nanoparticles, the intranasal administration, and the proposed mechanism of action in the lung. Mannan and dextran are amorphous homopolymers. The peptide R_p_H_q_C was conjugated to these polymers, where the straight chain represents the polymer backbone and the side beads represent the peptide sequence in the schematic. The polyplexes were formed through a solvent-free Flash Nano Complexation process using a custom-made dual-inlet vortex mixer (DIVM).

By mitigating these fundamental processes, TGF-β siRNA-based therapies offer the potential to attenuate or reverse pulmonary fibrosis. However, studies utilizing such RNA interference strategies in fibrotic lung disease have only recently emerged [20,[31], [32], [33], [34], [35]]. In this work, we developed a biomimetic and fully biodegradable peptide-mannan nanoparticle (PMNP) platform to enable intranasal delivery of TGF-β siRNA specifically targeting AMs in the lung (Fig. 1). Mannan was chosen as the backbone due to its monomeric unit, mannose, which is recognized and internalized by macrophages through CD206 scavenger receptors on the cell surface [36,37]. Notably, the C-type lectin domains (CTLDs) 4–8 of CD206 bind to mannose termini on carbohydrate molecules [[38], [39], [40]]. Furthermore, mannan's water-soluble nature supports an organic solvent–free nanoparticle manufacturing process, simplifying production. By exploiting mannan's ability to direct cargo towards macrophages, this design aims to deliver TGF-β siRNA to AMs for the treatment of lung fibrosis. To bind the negatively charged siRNAs and facilitate their subsequent intracellular release, mannan was conjugated to a peptide sequence containing positively charged arginine (R) residues for nucleic acid binding and cell penetration [[41], [42], [43]], as well as histidine (H) residues, whose imidazole groups facilitate endo/lysosomal escape through a proton-sponge effect [[44], [45], [46]]. Based on our previous studies, which identified optimal peptide sequences for high transfection efficiency and low toxicity, we focused on two peptide sequences R3H7C and R3H8C, each containing three arginine, seven or eight histidine, and one cysteine as the linker [[47], [48], [49]]. In this study, all polyplexes of peptide-polysaccharide and RNA were prepared through a solvent free method, through flash mixing in a custom-made dual-inlet vortex mixer (DIVM) integrated with computer-controlled syringe pumps (Fig. 1). The physicochemical properties of the resulting nanoparticles, including particle size distribution, morphology and structure, zeta potential, and encapsulation efficiency, where comprehensively characterized. *In vitro* and *in vivo* studies were performed to assess gene transfection efficiency, cytotoxicity, macrophage targeting, and therapeutic efficacy in attenuating and reversing fibrosis progression.

## 2 Results and discussion

### 2.1 Physicochemical properties of the PMNPs

**Fig. 2:**
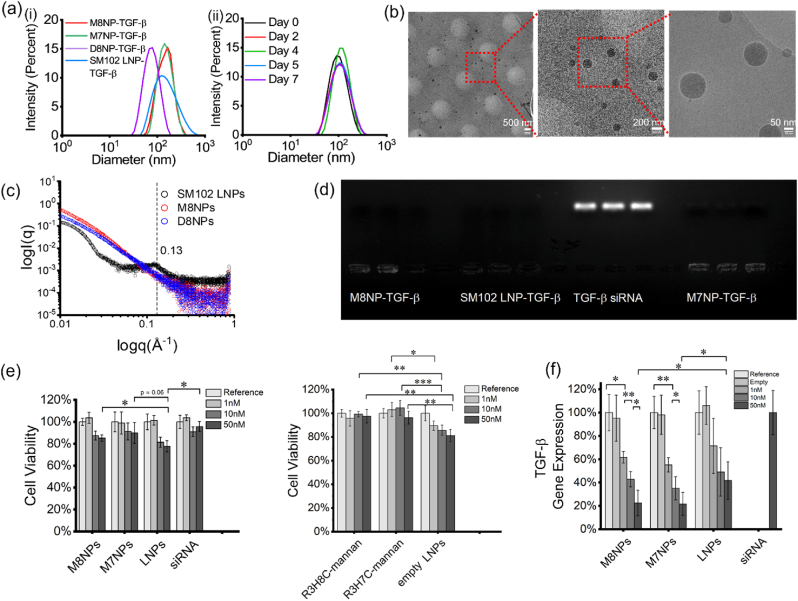
**Characterization of PMNP and SM-102 LNP physicochemical properties, cytotoxicity, and gene transfection efficiency *in vitro*.** (a) DLS analysis showing intensity-weighted size distribution of (i) M8NP-TGF-β, M7NP-TGF-β, D8NP-TGF-β, and SM-102 LNPs-TGF-β, and (ii) one-week colloidal stability of M8NP-TGF-β. (b)Cryo-TEM images of M8NP-TGF-β. (c) SAXS measurements of M8NP, D8NP, and SM-102 LNP. (d) Gel electrophoresis analysis demonstrating TGF-β siRNA loading efficiency in PMNPs and LNPs. (e) BMDM cell viability treated with P8NP-TGF-β, P7NP-TGF-β, and SM-102-LNP-TGF-β, and equivalent amount of free R3H8C-mannan, R3H7C-mannan, and empty LNPs. (f) Relative TGF-β gene expression in BMDMs treated with M8NP-TGF-β and M7NP-TGF-β with N/P ratio of 2 and SM-102 LNP-TGF-β with N/P ratio of 10, and gene expression in cells treated with empty NPs, and free siRNA. Statistical analysis was performed using One-way ANOVA for multiple-group comparisons and unpaired one-tailed t-tests for planned pairwise comparisons (n = 4). ∗p < 0.05, ∗∗p < 0.005, and ∗∗∗p < 0.0005.

Mannan and dextran were modified with the bifunctional linker divinyl sulfone (DVS), followed by conjugation with the peptide (Fig. S1). Mannan, dextran, the intermediate products, and the final conjugates were characterized by H^1^NMR in D_2_O (Figs. S2 and S3) and analyzed for molecular weight using matrix-assisted laser desorption/ionization (MALDI) mass spectroscopy (Fig. S4). An N/P ratio of 2 was selected for the formulation of TGF-β siRNA-loaded-peptide-mannan and peptide-dextran nanoparticles, based on our previous studies optimizing gene silencing efficacy while minimizing cytotoxicity [47,48]. The abbreviated names, compositions, hydrodynamic diameters, and zeta potentials of the nanoparticles are summarized in Table 1. DLS measurements showed that the PMNPs maintained an average hydrodynamic diameter below 200 nm, with only slightly broader distribution after one week of storage in DI water at room temperature (Fig. 2a), indicating good colloidal stability. The corresponding DLS correlation curves are provided in the Supplementary Material Fig. S5. M8NPs and D8NPs became much larger in PBS buffer within 30 min at 37^o^C, whereas they remained more stable in saline (Fig. S6). No buffer was added during intranasal administration. Due to the presence of an additional histidine in the side chain, M8NPs were slightly larger and exhibits marginally higher zeta potential than M7NPs. D8NPs exhibited smaller hydrodynamic diameters than M8NPs, likely due to the lower molecular weight of dextran (Mw ∼15-20 kDa) compared with mannan (Mw ∼34-62.5 kDa) (Fig. S4). All nanoparticles in this study exhibited a slightly positive zeta potential, reflecting moderately positive surface charges that facilitate cellular uptake while minimizing cytotoxicity [50]. The positive surface charge primarily arises from arginine (–NH–C(NH_2_)_2_^+^) residues in the peptide segment. The low N/P ratio of 2 for M8NPs, M7NPs, and D8NPs ensures only a slight excess of cationic groups relative to the negatively charged phosphate groups of siRNAs. In contrast, SM102-based lipid nanoparticles require much higher N/P ratio to achieve efficient encapsulation, with the positive charge arising from partial protonation of the ionizable lipid at neutral pH. Cryo-TEM Imaging (Fig. 2b) showed that M8NPs were predominantly spherical complexes with well-defined boundaries. The micrographs also revealed a homogeneous contrast without any evidence of ordered-phase structures, consistent with the absence of diffraction peaks in the SAXS profile. In contrast, the SAXS curve of SM102 LNPs exhibited a broad diffraction peak at $q\approx 0.13\;A^{-1}$, indicating the presence of lipid/RNA ordered phase (Fig. 2c). The amorphous internal structure of M8PN likely have higher configurational entropy and weaker intermolecular packing [51]. This disordered packing arises from the random distribution of peptides conjugated along the amorphous mannan polymer backbone.

**Table 1:** Nanoparticle abbreviation, composition, and their corresponding nanoparticle size and zeta potential.

Abbreviated name	Composition and description	DLS intensity average hydrodynamic diameter (nm)	PDI	Zeta potential (mV)
M8NP-TGF-β	R3H8C-mannan with TGF-β siRNA loaded nanoparticles at N/P ratio of 2	160.1 ± 11.75	0.117	+10.3
D8NP-TGF- β	R3H8C-dextran with TGF-β siRNA loaded nanoparticles at N/P ratio of 2	66.8 ± 0.65	0.148	+3.1
M7NP-TGF-β	R3H7C-mannan with TGF-β siRNA loaded nanoparticles at N/P ratio of 2	146.5 ± 15.04	0.230	+7.6
SM102 LNP-TGF-β	Moderna Ionizable lipid nanoparticles with 50 mol% SM-102, 10 mol% DSPC, 1.5 mol% DMG-PEG(2k), 38.5 mol% cholesterol loaded with TGF-β siRNA at N/P ratio of 10	121.6 ± 13.5	0.197	+10.8
M8NP-poly(I:C)	R3H8C-mannan with poly(I:C) loaded nanoparticles at N/P ratio of 2	186.1 ± 4.3	0.131	+7.2
D8NP-poly(I:C)	R3H8C-mannan with poly(I:C) loaded nanoparticles at N/P ratio of 2	65.47 ± 7.4	0.163	+4.1

Gel retardation electrophoresis verified nearly complete siRNA encapsulation in the nanoparticles (Fig. 2d), as no detectable fluorescence was observed from the nanoparticle suspensions. In addition, the encapsulation efficiency of M8NPs was quantified as 98.36% using the RiboGreen RNA Assay Kit with the calibration presented in Fig. S7.

### 2.2 *In vitro* toxicity and gene transfection

An MTT cytotoxicity assay was conducted to assess the biocompatibility of the PMNPs and compared to benchmarking SM-102 LNPs in bone marrow-derived macrophages (BMDMs). Untreated cells served as the negative control. Both siRNA loaded nanoparticles and carrier materials (peptide-mannan and empty LNPs) were included as controls to evaluate inherent material-associated toxicity. Three TGF-β siRNA concentrations (1 nM, 10 nM, and 50 nM) were tested, with corresponding peptide-mannan and LNP concentrations adjusted accordingly. PMNP and LNP suspensions were diluted 1:10 in DMEM/F12 without phenol red cell-culture medium before treatment. As shown in Fig. 2e, in the absence of siRNA, both R3H7C-mannan and R3H8C-mannan exhibited higher cell viability compared to empty SM102 LNPs toward BMDM cells at all tested concentrations. In contrast, lipids of SM-102 can self-assemble into nanoparticles regardless of siRNA presence, leading to similar cytotoxicity between empty and siRNA-loaded LNPs. The siRNA loaded nanoparticles (PMNPs and LNPs) demonstrated dose-dependent toxicity within the tested concentration range, with higher concentrations correlating with increased cytotoxicity. All PMNP-TGF-β displayed a similar dose-dependent toxicity trend but consistently lower than the SM-102 LNP-TGF-β across all concentrations, indicating a more favorable safety profile. At low concentrations (1 nM and 10 nM), PMNP-treated cells maintained viability close to 100%, with fluctuations attributable to minor experimental variation, consistent with the natural origin of the materials.

The *in vitro* transfection experiment was conducted to assess the efficiency of PMNPs and LNPs in silencing TGF-β mRNA expression in BMDM cells. The results are presented in Fig. 2f. Both M8NP-TGF-β and M7NP-TGF-β demonstrated better or comparable gene silencing efficiency compared to SM-102 LNP-TGF-β across all siRNA concentrations. No significant difference was observed between TGF-β loaded M8NPs and M7NPs. Gene knockdown efficiency was dose-dependent, with higher siRNA concentrations correlating with greater reductions in TGF-β mRNA expression. At 50 nM siRNA, M7NP-TGF-β and M8NP-TGF-β reduced TGF-β mRNA expression to 22.5% and 21.7%, respectively, compared to the control. At 10 nM, M8NP-TGF-β reduced mRNA expression to 42.9%, while M7NP-TGF-β achieved a slightly more knockdown at 35.1%. At the lowest concentration (1 nM), M8NP-TGF-β reduced mRNA expression to 61.7%, while M7NP-TGF-β achieved 55.2%. In comparison, SM-102 LNP-TGF-β exhibited less gene knockdown efficiency at all siRNA concentrations. At 50 nM, SM-102 LNP-TGF-β reduced TGF-β mRNA expression to approximately 40%, nearly twice the residual expression levels observed with PMNP-TGF-β. Similarly, LNPs showed limited gene silencing at 10 nM and 1 nM, with significantly higher mRNA expression compared to PMNP-treated cells. Neither R3H8C-mannan nor R3H7C-mannan (without siRNA) induced TGF-β mRNA reduction, confirming that the observed knockdown was attributed to the siRNA delivery function of the PMNPs. The enhanced gene silencing performance of the PMNPs is likely due to the rational design of the peptide component, which includes arginine for macrophage cell penetrating and histidine for endosomal escape and gene release via the proton-sponge effect [[44], [45], [46]]. Collectively, these findings demonstrate that PMNPs are less cytotoxic and more effective delivery system for TGF-β siRNA compared to for benchmark SM-102 LNPs, with improved cytocompatibility and gene transfection performance in macrophages.

### 2.3 Endo/lysosomal escape events visualization

**Fig. 3:**
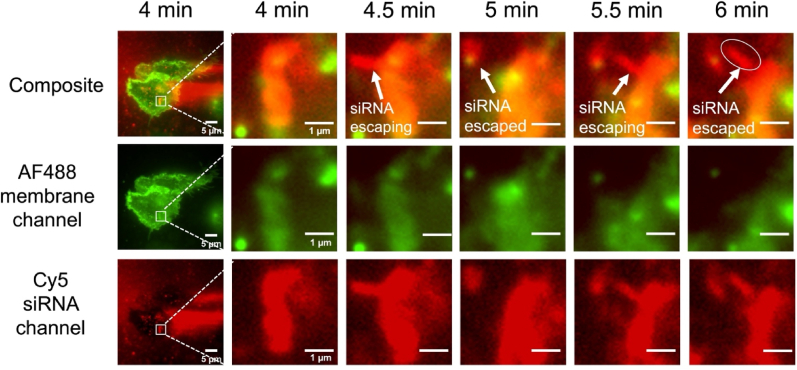
**Representative HILO images illustrating siRNA escape through endosomal recycling tubules**. The top row presents composite images of the membrane channel (green, second row) and Cy5-labeled siRNA channel (red, third row). Images within each column correspond to the same time point and the identical field of view. The second and subsequent columns display magnified view of the region highlighted in the first column. Scale bar: 5 μm (first column); 1 μm (subsequent columns).

The endo/lysosomal escape of PMNPs was investigated using total internal reflection fluorecence (TIRF) microscopy operated in the higly inclined and lamnated optical sheet (HILO) mode. Macrophage (RAW264.7) cell membranes were labeled with Alexa Fluor 488 (AF488) using the pan-membrane-protein labeling technique prior to treatment with M8NPs encapsulating Cy5-labeled siRNA for 30 min. Cells were then imaged in the presence of the Cy5-labeled siRNA in the HILO mode. Two distinct endo/lysosomal escape events were observed between 4 and 6 min after the start of imaging. In the zoom-in region shown in Fig. 3, overlapping AF488 (plasma membrane label) and Cy5 (siRNA label) signals were first observed at 4 min, indicating siRNA internalization through membrane-mediated endocytosis and subsequent accumulation within endo/lysosomes. At 4.5 min, a tube-like protrusion of Cy5-siRNA emerged with the absence of the membrane signal, suggesting partial release of siRNA from endo/lysosomes. By 5 min, the Cy5-siRNA signal had fully separated from the main endo/lysosome, with only a punctate membrane signal, suggesting possible siRNA escape accompanied by partial retention of endo/lysosomal membrane fragments. At 5.5 min, a second tube-like extrusion of Cy5-siRNA emerged, again with a diminished AF488 signal, implying progressive endo/lysosomal membrane disruption. By 6 min, the tubular Cy5 signal had fully dissociated from the main endo/lysosome, confirming successful escape of siRNA into the cytoplasm. The complete 1 h time-lapse composite cellular imaging is provided in Movie S1, displayed at 5 frames per second. Such tubular extrusion of RNA during escape is consistent with the previous observation by P. Prasath et al. [52], described as endosomal recycling tubule–mediated release. Nevertheless, more specific labeling will be required in the future to clearly distinguish between endosomal and lysosomal escape.

### 2.4 Internalization mechanism of PMNPs by macrophages

**Fig. 4:**
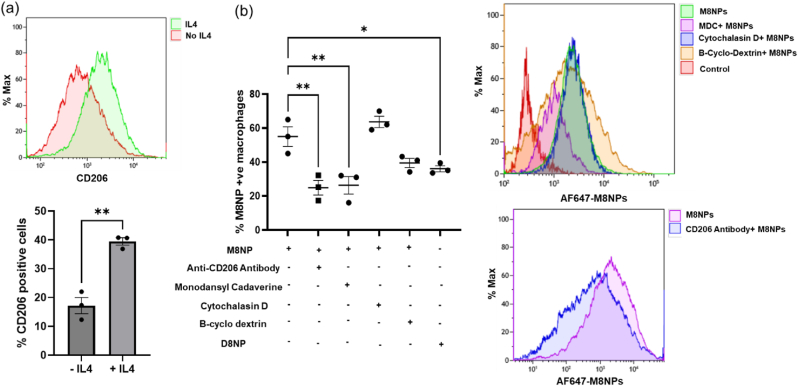
**M8NP-TGF-β nanoparticles are internalized via receptor-mediated endocytosis in M2 macrophages**. (a) IL4 induces CD206 expression in bone marrow derived macrophages. (b)Treatment with monodansyl cadaverine (MDC), an inhibitor of receptor-mediated endocytosis, significantly reduced the M8NP internalization in IL-4-stimulated M2 macrophages. In contrast, treatment with the pinocytosis inhibitor cytochalasin D did not inhibit M8NP internalization. Similarly, the Caveolae-mediated endocytosis inhibitor β-cyclodextrin had only a minimal inhibitory effect on M8NP internalization. CD206-mediated uptake was further confirmed by pretreatment with an anti-CD206 antibody, which significantly inhibited M8NP internalization. In comparison, D8NPs exhibited lower levels of internalization in IL-4-stimulated M2 macrophages. (Left) Quantification of mean fluorescence intensity (MFI) by flow cytometry. (Right) Representative overlaid flow cytometry histograms comparing nanoparticle uptake with and without MDC treatment. Statistical analysis was performed using one-way ANOVA. ∗p < 0.05 and ∗∗p < 0.005.

Understanding the cellular internalization mechanisms of nanoparticle-based therapeutics is essential for effective and selective siRNA delivery. In this study, our objective is to efficiently deliver siRNA to the profibrotic macrophages, which have elevated expression of the CD206 receptor. To model this phenotype *in vitro*, mouse bone marrow derived macrophages (BMDMs) were treated with IL4 (20 ng/ml) for 48 h to induce CD206 expression (Fig. 4a). To determine whether PMNP uptake occurs through receptor-mediated endocytosis or phagocytosis, we conducted an *in vitro* study using a monodansyl cadaverine (MDC), an established inhibitor of clathrin-mediated endocytosis. Flow cytometry analysis revealed that pretreatment with MDC significantly reduced PMNP internalization by BMDMs (Fig. 4b). In contrast, treatment with cytochalasin D, a pinocytosis inhibitor used as a negative control, didn't affect nanoparticle uptake. Similarly, use of β-cyclodextrin, a caveolae-mediated endocytosis inhibitor, had only a marginal effect on the M8NP internalization (Fig. 4b). These findings confirm that PMNP uptake is primarily mediated by receptor-dependent endocytosis rather than phagocytosis. The incorporation of mannan to form the particles with mannan on the surface is expected to promote specific binding to CD206 receptor. Consistent with this hypothesis, pretreatment of macrophages with an anti-CD206 antibody resulted in a significant reduction in M8NP internalization (Fig. 4b). In contrast, D8NPs had substantially lower levels of cellular internalization in IL-4-stimulated M2 macrophages. Together, these findings support a CD206-mediated uptake mechanism and demonstrate the advantage of mannan functionalization for targeted siRNA delivery to profibrotic macrophages.

### 2.5 *In vivo* targeting of PMNPs

**Fig. 5:**
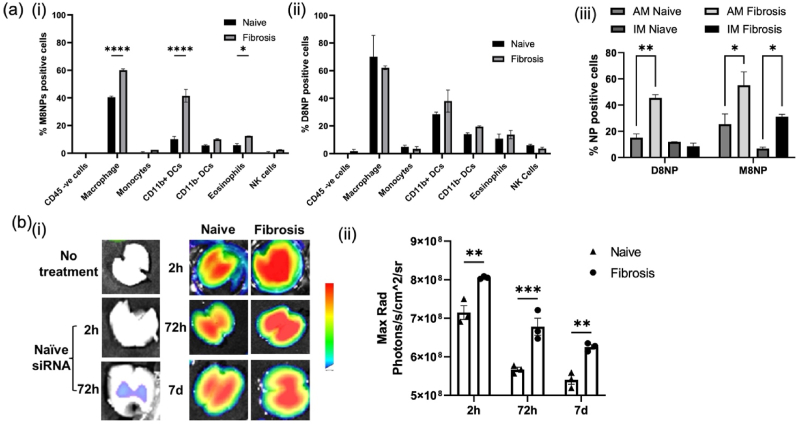
***In vivo* distribution of AF647-labeled M8NPs and D8NPs following intranasal administration.** (a) Flow cytometry analysis of nanoparticle distribution of (i) M8NPs (ii) D8NPs across lung non-immune and immune cells in naïve and fibrotic mice (day 15 post-bleomycin) (n = 3 animals). Macrophages exhibited the highest uptake of M8NPs. The gating strategy of immune cells was as follows: macrophages: CD45+Gr1-CD64^+^; monocytes: CD45+Ly6G-MHCII-CD64^+^CD11b+; dendritic cells: CD45+Ly6G-CD64-MHCII+; eosinophils: CD1b-CD45+Ly6G-CD64-MHCII-CD11b+; NK cells: CD45+Ly6G-MHCII-CD64^−^CD11b+. The cell populations were gated first, after which the percentage of nanoparticle-positive cells was calculated within each gate. **(iii)** Internalization of D8NPs and M8NPs by alveolar macrophages and interstitial macrophages under naïve and fibrotic conditions. The full gating strategy and corresponding flow cytometry histoplots are shown in Figure S10 and Figure S11. (b) *Ex vivo* IVIS imaging of lungs harvested from naïve and fibrotic mice at 2h, 72 h, and 7 days post-intranasal administration of M8NPs. (i) Representative IVIS images comparing free siRNA in fibrotic mice (left column) with siRNA encapsulated in M8NPs in naïve and fibrotic mice (right two column). (ii) Quantitative analysis of photon radiance from the lung tissues following M8NP intranasal delivery demonstrates prolonged nanoparticle retention in fibrotic lungs. Statistical analysis was performed using one-way ANOVA. ∗p < 0.05, ∗∗p < 0.005, and ∗∗∗p < 0.0005.

To assess the targeting efficiency of PMNPs, AF647-labeled PMNPs loaded with poly (I:C), serving as a surrogate for siRNA, were administered intranasally to both naïve and bleomycin-induced fibrotic mice. The size and zeta-potential of the poly (I:C) loaded PMNPs are similar as those TGF-β siRNA loaded PMNPs (Table 1 and Fig. S8). Nanoparticle biodistribution across lung cells populations was analyzed by flow cytometry after gating of the relevant cell population. D8NPs, which replaced the mannan backbone with dextran, were used as a comparison control. The CD45 negative cells including all non-hematopoietic cells such as fibroblasts, endothelial cells, and epithelial cells showed negligible nanoparticle internalization. Under fibrotic conditions, the M8NPs showed minor level (3%) of uptake by CD45 negative cells, suggesting absence of any non-specific effects on healthy cells (Fig. 5a (i)). In contrast, M8NPs were predominantly internalized by macrophages, with alveolar macrophages (AMs) showing the highest uptake (>50%) (Fig. 5a (iii)). In fibrotic lungs, interstitial macrophages (IMs), which are known to be profibrotic, exhibited significantly higher internalization (∼30%) compared to naïve mice (∼7%) (Fig. 5a (iii)). This enhanced uptake in IMs is likely due to enhanced vascular permeability associated with fibrosis progression [53]. During fibrosis, the infiltrating monocyte-derived IMs undergo a transitional state [54], and are known to express higher CD206 as compared to the healthy ones [47]. CD206 is a key mannose receptor tared by PMNPs. This is further supported by the observation of significantly higher M8NP positive IMs in fibrotic mice compared to healthy mice (Fig. 5a (iii)). Approximately 40% of monocyte-derived dendritic cells (CD11b^+^ DCs) also internalized PMNPs, consistent with their CD206 expression and profibrotic role, whereas monocytes, dendritic cells, eosinophils, and NK cells exhibited minimal nanoparticle internalization, underscoring the macrophage-targeting specificity of the PMNPs. In contrast, D8NPs, which lack mannose targeting, showed no significant difference in the internalization between naïve and fibrotic mice (Fig. 5a (ii) and **(iii))** other than the alveolar macrophages**.** The profibrotic IMs also exhibited limited D8NP uptake. Overall, M8NPs demonstrated substantially higher internalization in fibrotic lungs compared to D8NPs, highlighting the importance of CD206-mediated targeting in the mannose-modified nanoparticle system. Free siRNA controls showed only ∼30% macrophage association, primarily in AMs. Internalization by IMs was about 10% (Fig. S9).

Clearance kinetics were assessed through *ex vivo* imaging of lungs collected at various time points post-administration of AF647-labeled M8NPs. The results revealed a significantly higher and prolonged accumulation of M8NPs in fibrotic lungs compared to naïve lungs, with detectable signals persisting up to 72 h post-delivery (Fig. 5b). Notably, M8NPs remained present for up to 7 days in fibrosis-affected lungs (Fig. 5b (ii)). Although the moderately positive surface charges of the particles (zeta-potential ∼ +10 mV) may contribute to muco-adhesion, preferential targeting of the fibrotic microenvironment appears to be the primary mechanism underlying the prolonged retention of M8NPs in fibrotic lungs. These findings underscore the potential of intranasally delivered PMNPs as a macrophage-targeted, long-retaining siRNA delivery platform for the treatment of pulmonary fibrosis.

### 2.6 Assessment of hepatotoxicity and kidney function

**Fig. 6:**
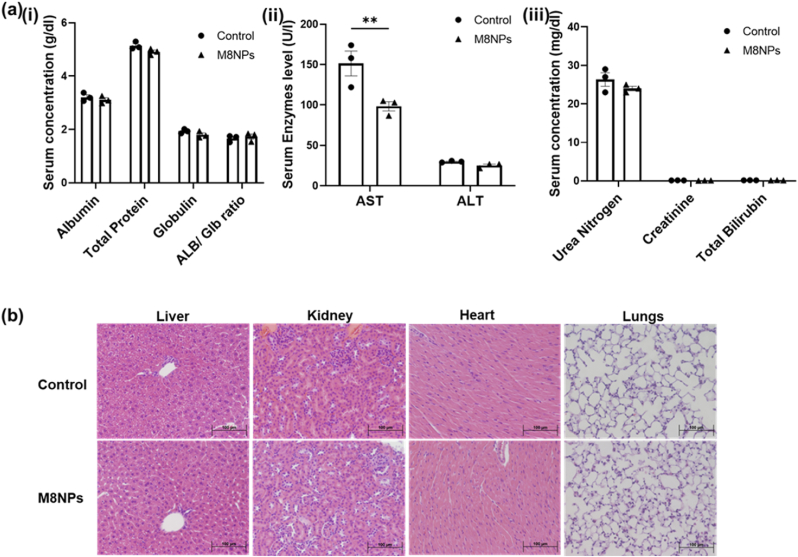
**Toxicity analysis** (a) Serum biochemistry analysis for the assessment of hepatotoxicity and nephrotoxicity after intranasal administration of M8NPS. Parameters analyzed include (i) albumin, globulin, total protein, and albumin/globulin ratio; (ii) liver enzyme alanine aminotransferase (ALT) and aspartate aminotransferase (AST); and (iii) kidney function markers urea nitrogen, creatinine and total bilirubin, (b) Histological analysis to assess toxicity of M8NPs.

Given the small intranasal dose of PMNP suspension administered, minimal systemic exposure and toxicity were anticipated. To evaluate potential adverse effects, serum biochemistry and histology were analyzed in naïve mice 72 h after M8NPs administration to assess hepatotoxicity or kidney function (Fig. 6). No significant difference in serum proteins levels, including albumin and globulin, indicating the absence of hepatic dysfunction (Fig. 6a (i)). Liver enzymes profiles also showed no evidence of toxicity. Although aspartate aminotransferase (AST) levels were slightly lower in the nanoparticle-treated group, other hepatic indicators, such as bilirubin, remained within normal ranges (Fig. 6a (ii) and (iii)**)**. Furthermore, no signs of renal toxicity were detected, as urea nitrogen levels were comparable to those of control animals (Fig. 6a (iii)). The results collectively suggest that intranasal administration of M8NPs does not induce hepatotoxicity or impair kidney function under the tested conditions. Further, the histology of different organs (liver, kidney, heart and lungs) showed no signs of toxicity after M8NPs treatment to mice (Fig. 6b). Only acute effects following two intranasal doses of PMNP-TGF-β siRNA were evaluated in this study. Comprehensive safety assessment, including higher doses and long-term evaluation of hepatic and renal function, will be necessary in future studies to support translational applications.

### 2.7 Therapeutic effect of TGF-β loaded PMNPs

**Fig. 7:**
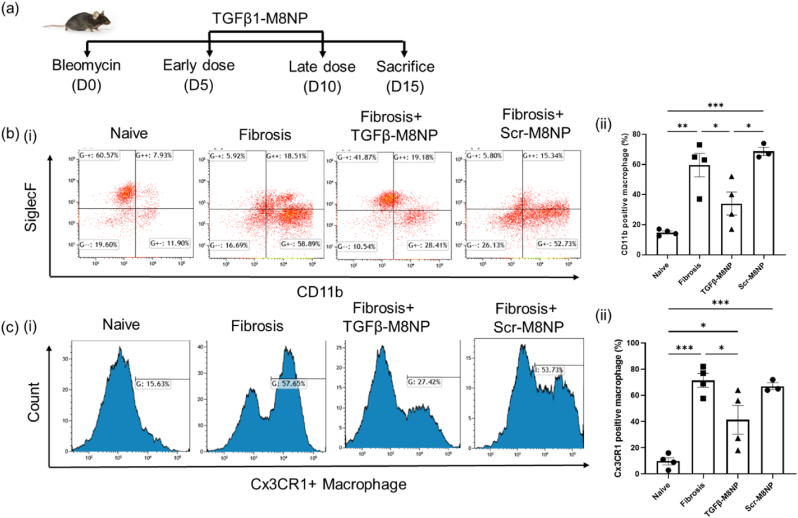
**M8NP-TGF-β modulates macrophage profile.** (a) Schematic of the therapeutic workflow for M8NP-TGF-β administration in the bleomycin model. (b) Lung macrophage profiles (CD45^+^ Gr1- CD64^+^) in naïve, fibrotic, M8NP-TGF-β-treated fibrotic mice, and M8NP-scrambled siRNA treated fibrotic mice. (i) Representative flow cytometry dot plots. (ii) Quantitative analysis, showing that M8NP-TGF-β treated mice prevented the depletion of tissue-resident AM and controlled the infiltration of Mo-AM as revealed by lesser CD11b positive macrophages. (c) Infiltration analysis of Cx3Cr1+ macrophages during fibrosis in Cx3cr1CreERT2 (tamoxifen-inducible Cx3cr1 monocyte-specific Cre recombinase) lineage mice. (i) Flow cytometry histograms showing reduced Tdtomato^+^ macrophages (gating strategy: CD45+Gr1-CD64+TDTomato+). (ii) Quantitative representation confirming reduced Mo-AM infiltration in M8NP-TGF-β treated lungs.

We next evaluated the therapeutic efficacy of M8NP-TGF-β in a bleomycin-induced lung fibrosis model over a 15-day period. M8NP-TGF-β NPs were administered intranasally on day 5 and day 10 post-bleomycin instillation, targeting the macrophages during the critical window of fibrotic progression. Following treatment, flow cytometry analysis revealed a reversal of the macrophage profile, characterized by a gradual reduction in the CD11b^+^ macrophage population and a restoration of Siglec-F^+^ CD11b^−^ macrophages, which are associated with lung homeostasis (Fig. 7b).

During fibrosis progression, the lung becomes heavily infiltrated with Mo-AMs, which secrete profibrotic cytokines such as TGF-β and PDGF-α, thereby perpetuating disease progression. Several studies, including our own, show that Cx3CR1 positive monocyte-derived macrophages retained in the fibrotic niche [32,54]. To assess the fate of infiltrating Mo-AMs, we utilized Cx3cr1CreERT2+; Tdtomatofl/fl genetic lineage-tracing mice, in which circulating monocytes are labeled as Tdtomato^+^. Flow cytometry analysis (with the gating strategy illustrated in Fig. S10) revealed a significant reduction in Cx3CR1^+^ macrophages following M8NP-TGF-β treatment (Fig. 7c), confirming the efficacy of M8NP-TGF-β to inhibit TGF-β. Blocking TGF-β in alveolar macrophages disrupts the profibrotic cytokine milieu, preventing further infiltration of Mo-AMs and halting disease progression.

**Fig. 8:**
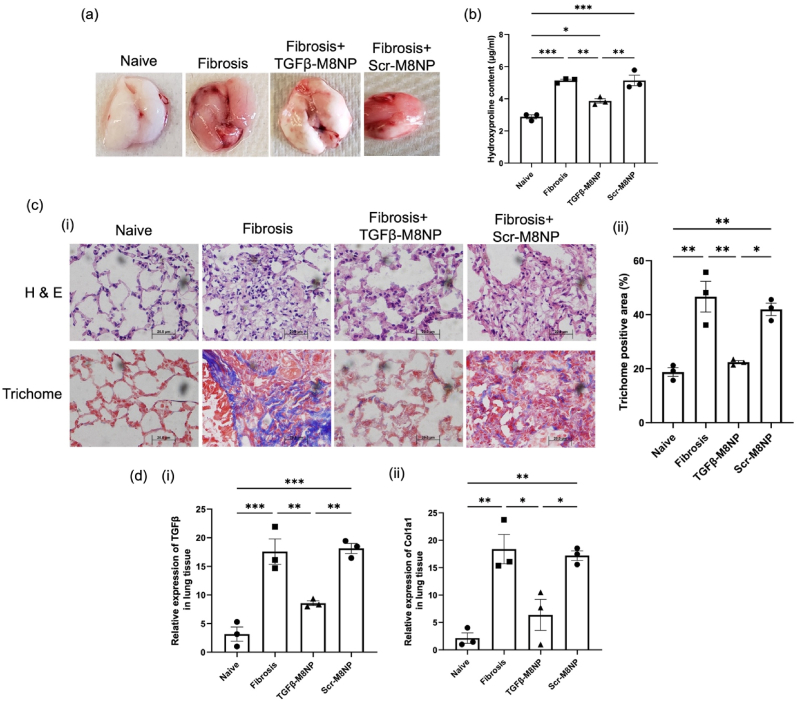
**M8NPs-TGF-β nanoparticles attenuate and reverse the progression of lung fibrosis by reducing collagen deposition and profibrotic gene expression.** (a) Representative lung micrographs comparing lung lesions in naïve, bleomycin-induced fibrotic, M8NP-TGF-β and M8NP-scrambled siRNA treated mice at day 15 post-instillation. (b) Hydroxyproline assay showing collagen content in lung tissues across the three groups. (c) Histological assessment: (i) H&E and Trichrome staining of lung sections from naïve, fibrotic, M8NP-TGF-β and M8NP-scrambled siRNA treated mice. (ii) Quantification of Trichrome stained areas using ImageJ software. (d) Quantitative PCR analysis of gene expression in lung tissues: (i) TGF-β and (ii) Collagen1 a1 mRNA levels in naïve, fibrotic, M8NP-TGF-β, and M8NP-scrambled siRNA treated groups. Statistical analysis was performed using One-way ANOVA. ∗∗p < 0.005, ∗∗∗p < 0.0005, and ∗∗∗∗p < 0.0001.

To further assess fibrosis resolution, we evaluated the overall fibrotic burden using histological analysis and hydroxyproline assays. Gross anatomical inspection of lung tissues revealed a visible reduction in fibrotic mass following M8NP-TGF-β treatment compared to untreated fibrotic controls and M8NP-scrambled siRNA treated mice (Fig. 8a). Quantitative hydroxyproline assays confirmed a significant decrease in collagen deposition in the lungs of M8NP-TGF-β treated mice (Fig. 8b). H&E and Trichrome staining revealed a marked reduction in fibrosis in treated lungs compared to controls (Fig. 8c (i)). Image-based quantification of Trichrome-positive areas confirmed statistically significant reductions in collagen-rich fibrosis, approaching levels observed in naïve animals (Fig. 8c (ii)). In addition to structural improvements, the treatment also led to a downregulation of profibrotic gene expression. Quantitative PCR analysis of lung tissues showed a significant reduction in both TGF-β and Collagen a1 in the lung tissues of the M8NP-TGF-β treated animals compared to fibrotic control and M8NP-scrambled siRNA treated animals (Fig. 8d (i) and **(ii**). During pulmonary fibrosis, macrophages produce profibrotic growth factors and chemokines that activate type II alveolar epithelial cells, leading to hyperplasia and myofibroblasts, which are responsible for excessive collagen production [55]. Thus, modulation of macrophages can prevent the reprogramming of the lung tissue and interrupt this pathological cascade. Collectively, these findings demonstrate that targeting TGF-β in alveolar macrophages not only reduces profibrotic cytokine secretion but also disrupts the macrophage–fibroblast signaling axis, thereby mitigating fibrosis progression.

### 2.8 Conclusions

In this study, we successfully designed and synthesized a peptide-mannan nanoparticle (PMNP) platform for siRNA delivery, specifically targeting pro-fibrotic macrophages associated with fibrosis. The continuous, scalable process of producing the RNA-loaded PMNPs is self-assembly and solvent-free, resulting in stable nanoparticles that remain intact at room temperature storage and nebulization conditions. Our primary objective was to selectively target pro-fibrotic monocyte-derived alveolar macrophages, a key driver of fibrosis progression, via non-invasive airway delivery. During pulmonary fibrosis, Mo-AMs displace long-term resident alveolar macrophages and become the dominant macrophage population in the airways. We demonstrated that intranasally administered PMNPs preferentially internalized by alveolar macrophages and that delivery of PMNP-TGF-β effectively modulated the pro-fibrotic macrophage phenotype, reduced profibrotic signaling, and halted disease progression. Compared to systemic injections, intranasal administration offers a patient-friendly, non-invasive alternative that enhances local lung bioavailability while minimizing off-target effects. Importantly, two doses of PMNP-TGF-β siRNA produced measurable reduction in fibrosis markers and histological improvement, while restoring macrophage population to near-normal level by day 15, indicating that prolonged TGF-β suppression is not required for therapeutic efficacy. This localized and transient TGF-β silencing strategy may reduce the long-term risks associated with immune dysregulation. Together, these findings highlight the therapeutic potential of PMNP-based airway gene delivery platforms in the treatment of pulmonary fibrosis. The approach established here lays the groundwork for future advancements in targeted gene therapies for respiratory diseases.

## 3 Materials and methods

### 3.1 Materials

Mannan (Mw 34k∼62.5k) from saccharomyces cerevisiae, divinyl sulfone (DVS), 4-(dimethylamino) pyridine (DMAP), deuterium oxide (99.9%), tris (2-carboxyethyl) phosphine hydrochloride (TCEP-HCl), nuclease-free water, sodium acetate, dextran (20k), and hydroxyproline assay were purchased from Sigma Aldrich (St. Louis, MO). Dimethyl sulfoxide (DMSO), 2-propanol, ethanol (200 proof reagents), agarose, streptomycin−penicillin, trypsin−EDTA, ROCHE MTT Cell Proliferation Kit, ethidium bromide, dialysis tubing 100∼500 MWCO, and gel loading dye were purchased from VWR International (Radnor, PA). The peptides RRRHHHHHHHHC (R3H8C, >95% purity), and RRRHHHHHHHC (R3H7C, >95% purity) were purchased from GenScript (Piscataway, NJ). GAPDH reverse and forward primers were purchased from Integrated DNA Technologies, Inc (Coralville, IA). Zeba Spin Desalting Columns (7k MOCO), RiboGreen RNA Assay Kit, PowerUp SYBR Green Master Mix, High-Capacity cDNA Reverse Transcription Kit, DMEM/F-12, CHCA MALDI Matrix, and fetal bovine serum (FBS), Alexa Fluor 647 Dye (AF647), phosphate-buffered saline (PBS, pH = 7.4) were purchased from ThermoFisher Scientific (Waltham, MA). TGF-β siRNA and Cy5-labeled TGF-β siRNA were purchased from Horizon discovery (Cambridge, UK). CD45-AF700, Gr-1-BV605, CD64-PECy7, SiglecF-BV421, CD11b-AF594, and CD206-AF488 were purchased from Biolegend. Eight-well chambered cover glasses (Sterile, No. 1, C8-1-N) were ordered from Cellvis.

### 3.2 Conjugation of peptides with mannan and dextran

Mannan (100 mg, 0.1eq) was dissolved in 15 mL of DMSO and heated to 70 °C for 1 h to ensure complete dissolution. Subsequently, 7.54 mg (1 eq in terms of repetitive structure) of DMAP and 1.854 mL (30 eq) of DVS were added to the mixture. The reaction was stirred for 28 h at 25 °C covered with aluminum foil and maintained in an oil bath. After the reaction, the mixture was filtered through DMSO-resistant filter paper, and the filtrate was added dropwise into 85 mL of diethyl ether. The resulting suspension was centrifuged at 10,000×*g* for 30 min to remove the supernatant. The remaining solid was redissolved in 5 mL of DMSO and then precipitated again by adding 85 mL of ethanol. This centrifugation and ethanol-washing step was repeated three times to remove residual DVS. The purified product, mannan-DVS, was redissolved in water, filtered to remove any insoluble materials, and finally lyophilized. ^1^H NMR in D_2_O confirmed that the degree of substitution (DS) with DVS ranged from 21 to 25% (Fig. S2). For the peptide-polysaccharide conjugation, R3H8C or R3H7C (50 mg) was dissolved in 100 μL of DI water. TCEP-HCl (1.13 mg, 0.2 eq) was added to the solution and incubated at room temperature for 15 min to reduce disulfide bonds that were formed during storage. The pH was adjusted to 6-6.5 by adding NaOH solution. To generate peptide-mannan conjugates, 13.8 mg of the mannan-DVS was dissolved in 200 μL of deionized water and mixed with the peptide solution. The pH of the mixture was maintained in the range 6-6.5 at 37 °C under gentle shaking for four days. Following the reaction, the product was purified using 7 kDa molecular weight cutoff (MWCO) desalting column, subsequently frozen at −80 °C overnight, and lyophilized. R3H8C-dextran was synthesized following the same steps. Due to the lower molecular weights and reduced polydispersity of the dextran, the purification process of dextran-DVS was simplified. Using diethyl ether and ethanol for washing was sufficient without the filtration step in water. The final products, R3H8C-mannan, R3H7C-mannan, and R3H8C-dextran were characterized by H^1^NMR in D_2_O (Figs. S2 and S3) and analyzed for molecular weight using MALDI Mass Spectroscopy (Fig. S4).

### 3.3 Preparation of PMNPs and LNPs

All nanoparticles were prepared using a custom-made dual-inlet vortex mixer (DIVM) integrated with computer-controlled syringe pumps. The R3H8C-mannan, R3H7C-mannan or R3H8C-dextran (280 nmol/mL based on repeating units) and TGF-β siRNA (10 nmol/mL) were each dissolved in RNase-free water of same volume. The two solutions were loaded into two 1 mL syringes, which were connected to a DIVM. The solutions were then simultaneously injected through the DIVM at a total flow rate of 40 mL/min. SM-102 LNPs was generated by mixing the organic solution containing 1680 nmol/mL SM-102, 336 nmol/mL DSPC, 50.4 nmol/mL DMG-PEG (Mw 2 kDa), and 1293.6 nmol/mL cholesterol dissolved in ethanol and the aqueous solution containing TGF-β siRNA dissolved in 50 mM pH 5.5 sodium acetate buffer at an N/P ratio of 10 with 40 mL/min total flow rate. The volume ratio of organic stream to aqueous stream is 1:3. The collected SM-102 LNP suspension was dialyzed against 100 times volume of the pH 7.4 PBS buffer with three changes of the buffer every 2 h, using 100-500 MWCO dialysis membrane.

### 3.4 Measurements of particle size, zeta potential, and structure

The hydrodynamic diameter and surface charge (zeta potential) of the nanoparticles were measured by dynamic light scattering (DLS) and zetasizer (Malvern Nano ZS, UK) with viscosity and light reflection index set to be 0.8872 cP and 1.330 from the default setting at 25 °C. Particle size distributions are reported as the intensity weighted. Nanoparticle morphology was examined using cryo-TEM (Thermo Scientific Glacios3 200 kV). The structure of the particles was further characterized using synchrotron small-angle X-ray scattering (SAXS) conducted at Sector 12 Chemical and Materials Science group, Advanced Photon Sources (APS) at Argonne National Laboratory.

### 3.5 Quantification of RNA encapsulation efficiency

Agarose gel electrophoresis was first used to assess gene loading efficiency. Agarose gel was prepared at 4% (w/v) in 1X TAE buffer. For each sample, 30 μL of the nanoparticle suspension was mixed with 10 μL of loading buffer and incubated for 15 min. Subsequently, 10 μL of the mixture was pipetted into each well with triplicates prepared for each sample. Gel electrophoresis was carried out in 1X TEA buffer at 110V for 60 min. Afterwards, the gel was stained with ethidium bromide (EtBr) at a final concentration of 0.5% mg/mL and visualized using the Azure C150 Gel Imaging System (Dublin, CA, USA). The fluorescence of free TGF-β siRNA was analyzed by ImageJ. No detectable free RNA signal was observed from the nanoparticle suspensions, indicating nearly complete encapsulation.

To quantify encapsulation efficiency, a RiboGreen RNA Assay Kit was employed. A series of TGF-β siRNA in TE buffer (20 ng/mL – 1000 ng/mL) were prepared in a 96-well plate for a calibration curve (Fig. S7). PMNP suspensions were ultrafiltrate with a 100K MWCO membrane. Equal volume of RiboGreen dye solution and filtrate were added to all wells, followed by incubation at room temperature for 5 min. Fluorescence intensity was measured using a SpectraMax M2 microplate reader (Molecular Devices, USA) with excitation at 480 nm and emission at 520 nm.

### 3.6 Macrophages cell-culture

Bone marrow-derived macrophages (BMDMs) were extracted from the long bones of female C57BL/6 mice. Bone marrow was flushed using Dulbecco's PBS using a 23G syringe needle, followed by filtration through a 70 mm cell strainer. Red blood cells were lysed and remaining monocytic cells were collected for differentiation. Cells were cultured toward macrophages in DMEM/F-12 (Dulbecco's Modified Eagle Medium/Nutrient Mixture F-12) supplemented with 10% of FBS, 5% of GlutaMAZ (100X), 1% penicillin-streptomycin, and 50 ng/mL macrophage colony stimulating factor (M-CSF). The cells were maintained at 37 °C in a humidified incubator with 5% CO_2_ and differentiated for 6 days, with a medium change on day 3. On day 6, BMDMs were harvested via gentle cell scraping for assay testing.

RAW264.7 cells were maintained in high-glucose DMEM supplemented with 10% FBS and 100 U/mL penicillin–streptomycin at 37 °C in a humidified incubator with 5% CO_2_. Cells were split upon reaching confluence.

### 3.7 Cytotoxicity assay

BMDMs were counted and seeded into 24-well plates at a density of 0.17 x 10^6^ cells per well and incubated for 24 h at 37 °C. After incubation, the culture medium was replaced with 450 μL of fresh DMEM/F-12 medium and 50 μL of nanoparticle suspensions (PMNPs or LNPs), resulting in final siRNA concentrations of 1 nM, 10 nM, and 50 nM. The cells were cultured with the nanoparticle treatments for an additional 24 h. The medium was then replaced with fresh DMEM/F-12 containing MTT stock solution, and the cells were incubated for 4 h. After incubation, 500 μL of MTT solubilization solution was added into each well to dissolve the crystals. The plates were incubated overnight. Absorbance of samples was measured at 570 nm with reference wavelength of 670 nm using a Tecan Infinite 200 microplate reader (Männedorf, Switzerland). As controls, solutions of siRNAs, peptide-mannan conjugates, and empty LNPs were included to evaluate toxicity effects of the individual components.

### 3.8 RNA interference assay

BMDMs were grown in a cell-culture flask, harvested, and counted using a hemocytometer. The cells were seeded into 24-well plates at a density of 0.17 x 10^6^ cells per well and 70% confluency and allowed to adhere overnight. The following day, the medium was replaced with fresh medium containing nanoparticles (PMNPs or LNPs) at three different TGF-β siRNA concentrations of 1 nM, 10 nM, and 50 nM. After 24 h of incubation, the medium was removed, and the cells were washed twice with PBS. Total RNA was extracted using TRIzol™ reagent, and cDNA was synthesized using a High-capacity cDNA Reverse Transcription Kit. The cDNA concentration was measured with a NanoDrop™ One spectrophotometer. RT-PCR was performed on the same day using PowerUp™ SYBR™ Master Mix. TGF-β gene expression levels were normalized to housekeeping gene GAPDH, and relative expression was calculated using the Livak (2^−ΔΔCt) method.

### 3.9 NHS-ester labeling of cells

The labeling technique was adapted from previously reported work [56]. Briefly, RAW264.7 cells were harvested and centrifuged at 300×*g* for 3 min at room temperature, and the supernatant was removed. The cell pellet was resuspended at a density of 2 × 10^5^ cells/mL in 500 μL NaHCO_3_ (0.1 M) and mixed with 20 μL Alexa Fluor 488 NHS ester (0.5 μg/μL in DMSO). The suspension was gently rotated in the dark at room temperature for 3 min, after which the reaction was quenched by adding 50 μL glycine (45 mg/mL in ultrapure water). Excess dye was removed by centrifugation (300×*g*, 3 min, room temperature), followed by two washing steps with complete medium. Finally, the labeled cells were resuspended in prewarmed complete medium, diluted as required for subsequent experiments.

### 3.10 Sample preparation and live-cell microscopy imaging

Labeled RAW264.7 cells were seeded at a density of approximately 80,000 cells per well and incubated for 2 h at 37 °C in a humidified atmosphere of 5% CO2. The medium was then replaced with complete medium containing siRNA M8NPs (50 nM), and cells were incubated for an additional 30 min before imaging.

Total-internal-reflectance fluorescence (TIRF) microscopy was used to perform imaging in the highly inclined and laminated optical sheet (HILO) mode using ND acquisition in Nikon NIS-Elements Advanced Research software (Version: 5.42.06) on an inverted Nikon Ti2E microscope equipped with a 100x/1.49 oil-immersion objective (CFI Apochromat TIRF 100XC). Time-lapse movies with 30 s time intervals were recorded using a Prime 95B sCMOS camera (Serial Number: A21C203010) at 16-bit. An integration time of 50 ms was used with 488 and 640 nm lasers at laser power densities of 13.55 and 69.43 W/cm^2^, respectively. Movie was collected for 1 h at room temperature. TIRF microscopy images were visualized and analyzed using ImageJ (Fiji) 1.54 p.

### 3.11 Cell internalization mechanism study

To investigate the cellular internalization mechanism of PMNPs, BMDMs were seeded in 12-well plates and polarized toward the M2 macrophages by treatment with IL-4 for 24 h, thereby upregulating CD206 receptor expression. After polarization, cells were pretreated with either a CD206 receptor–blocking agent or monodansylcadaverine (MDC), an inhibitor of clathrin-mediated endocytosis, or other inhibitors to evaluate potential uptake pathways involved in PMNP internalizations. After pretreatment, the inhibitors were applied for 15 min, followed by the addition of 50 nM AF647-labeled PMNPs for 30 min. For nanoparticle labeling, peptide-mannan conjugates were dissolved in DMSO and incubated with AF647 dye for 24 h to enable covalent fluorophore conjugation. The labeled conjugates were subsequently mixed with an aqueous phase containing poly(I:C) as a siRNA substitute to form the fluorescent nanoparticles. After 30 min of nanoparticle incubation, cells were washed, fixed, and analyzed via flow cytometry to quantify nanoparticle uptake.

### 3.12 Animal experiment

All animal experiments were conducted using C57BL/6 mice (Jackson Laboratory), with animals randomly assigned to experimental groups. All procedures were approved by the University of Illinois Animal Care and Use Committee and performed in accordance with institutional guidelines. Pulmonary fibrosis was induced using an established *in vivo* model. Briefly, mice were anesthetized and administrated bleomycin intratracheally at a dose of 0.015 U/20g body weight, and the animals were euthanized on day 15 post-instillation.

For lineage tracing experiments, Cx3cr1CreERT2 (tamoxifen-inducible Cx3cr1 monocyte-specific Cre recombinase) mice (Jax Cat. No. 021160), were crossed with Tdtomatofl/fl (Jax Cat. No 007909) mice to generate inducible Cx3cr1CreERT2+; Tdtomatofl/fl mice. Genotyping of mice strains was performed by standard PCR using the primers recommended on the Jackson Laboratory website (www.jax.org). To induce Cre recombination, Cx3cr1CreERT2 mice were administered tamoxifen (75 mg/kg body weight) via intraperitoneal injection, starting one week before bleomycin instillation and continuing every other day throughout the treatment.

### 3.13 *In vivo* uptake of PMNPs

The *in vivo* uptake of M8NPs by macrophages was evaluated in a bleomycin-induced pulmonary fibrosis mouse model on day 7 post-bleomycin challenge. Both C57Bl6 and Cx3cr1CreERT2+ Tdtomatofl/fl mice were administrated AF647-labeled PMNPs intranasally 2 h prior to euthanasia. Following euthanasia, lungs were perfused with PBS and processed for single-cell isolation. The resulting lung cell suspension was analyzed by flow cytometry to assess nanoparticle uptake.

### 3.14 Therapeutic effects of PMNP-TGF-β siRNA

To evaluate the therapeutic efficacy of M8NP-TGF-β, fibrotic mice received intranasal administrations of M8NPs containing 0.1 nmol Cy5-labeled TGF-β siRNA on days 5 and 10 post-bleomycin instillation. On day 15 post-instillation, the mice were sacrificed, and the lung tissues were collected for further analysis.

### 3.15 Flow cytometry analysis

Following euthanasia, lung tissues were collected, finely minced, and digested in 1X collagenase in DMEM media for 1 h. The resulting cell suspensions were filtered through a 70 μm cell strainer, followed by RBC lysis using a lysis buffer. The remaining cells were further filtered through a 40 μm filter to obtain a single cell suspension. For surface marker staining, cells were incubated with fluorophore-conjugated antibodies targeting CD45-AF700, Gr-1-BV605, CD64-PECy7, SiglecF-BV421, CD11b-AF594, and CD206-AF488 (all from Biolegend). Stained samples were analyzed using Flow Fortessa (BD Biosciences). Data was analyzed by using Kaluza Analysis 2.1 (Beckman). The gating strategy started with the selection of live, single cells followed by the identification of different cell types. For biodistribution analysis, the different immune cells were gated following our previously published protocol [32].

### 3.16 Histological analysis

For histological analysis, lungs from naïve, fibrotic, and PMNP-treated mice were perfused and fixed using 10 % buffered formaldehyde. Fixed tissues were processed for paraffin embedding and sectioned. Histological sectioning and staining, including Hematoxylin & Eosin (H&E) and Trichrome staining, were performed with the help of Research Histology Core (RHC) at the University of Illinois, Chicago. Quantification was conducted using ImageJ software.

### 3.17 Hydroxyproline assay

To assess the fibrotic burden and evaluate the therapeutic efficacy of the PMNP treatment in reducing the fibrotic burden, collagen content in lung tissue was quantified using a hydroxyproline assay, following the manufacturer's instructions. For each mouse, the entire left lobe of the lung was collected post-euthanasia.

### 3.18 Statistical analysis of experimental data

Statistical significance was determined using Prism v.9.1.1 (GraphPad Software). All experiments were independently repeated at least three times. Sample size and p-values are provided in the corresponding figures and captions.

## CRediT authorship contribution statement

**Bailin Feng:** Writing – review & editing, Writing – original draft, Visualization, Investigation, Formal analysis, Data curation. **Abhalaxmi Singh:** Writing – review & editing, Writing – original draft, Visualization, Methodology, Investigation, Formal analysis, Data curation, Conceptualization. **Yiqing Yang:** Writing – review & editing, Writing – original draft, Investigation, Formal analysis, Data curation. **Philana Phan:** Writing – review & editing, Writing – original draft, Investigation. **Han Xu:** Writing – review & editing, Writing – original draft, Visualization, Investigation. **Yuli Zhu:** Writing – review & editing, Writing – original draft, Investigation. **Jennifer Huang:** Writing – review & editing, Investigation. **Vrushank Sastry:** Investigation. **Zongmin Zhao:** Writing – review & editing, Supervision, Funding acquisition. **Ying S. Hu:** Writing – review & editing, Supervision, Methodology. **Gang Cheng:** Writing – review & editing, Writing – original draft, Visualization, Supervision, Methodology, Conceptualization. **Asrar B. Malik:** Writing – review & editing, Supervision, Conceptualization. **Ying Liu:** Writing – review & editing, Writing – original draft, Supervision, Project administration, Methodology, Funding acquisition, Formal analysis, Conceptualization.

## Ethics approval and consent to participate

All animal experiments were conducted using C57BL/6 mice (Jackson Laboratory), with animals randomly assigned to experimental groups. All procedures were approved by the University of Illinois Animal Care and Use Committee (approved animal protocol: ACC 23-105) and performed in accordance with institutional guidelines.

## Funding

This work was supported by the 10.13039/100008522University of Illinois Chicago (UIC) Chancellor's Translational Research Initiative (CTRI) grant to YL. AS was supported by 10.13039/100000002National Institutes of Health (NIH) P01 HL151327. Z.Z. acknowledges the support from 10.13039/100000002NIH/NHLBI (R21HL168650). P.P. acknowledges support from the 10.13039/100000002National Center for Complementary & Integrative Health of the National Institutes of Health under Award Number T32AT007533.

## Declaration of competing interest

The authors declare the following personal relationships which may be considered as potential competing interests: Asrar B. Malik is currently employed by Cell Biologics.
