# Supplementary material for: Polyplex of peptide-mannan and RNA for intranasal delivery of TGF-β siRNA in treatment of pulmonary fibrosis
Source: Bioact Mater. 2026 Feb 6;61:20–33. doi: 10.1016/j.bioactmat.2026.02.006 (PMC12907503; doi:10.1016/j.bioactmat.2026.02.006)
Supplement: Multimedia component 1 [file mmc1.docx]

**Supporting Information**

**Polyplex of Peptide-Mannan and RNA for Intranasal Delivery of TGF-β siRNA in Treatment of Pulmonary Fibrosis**


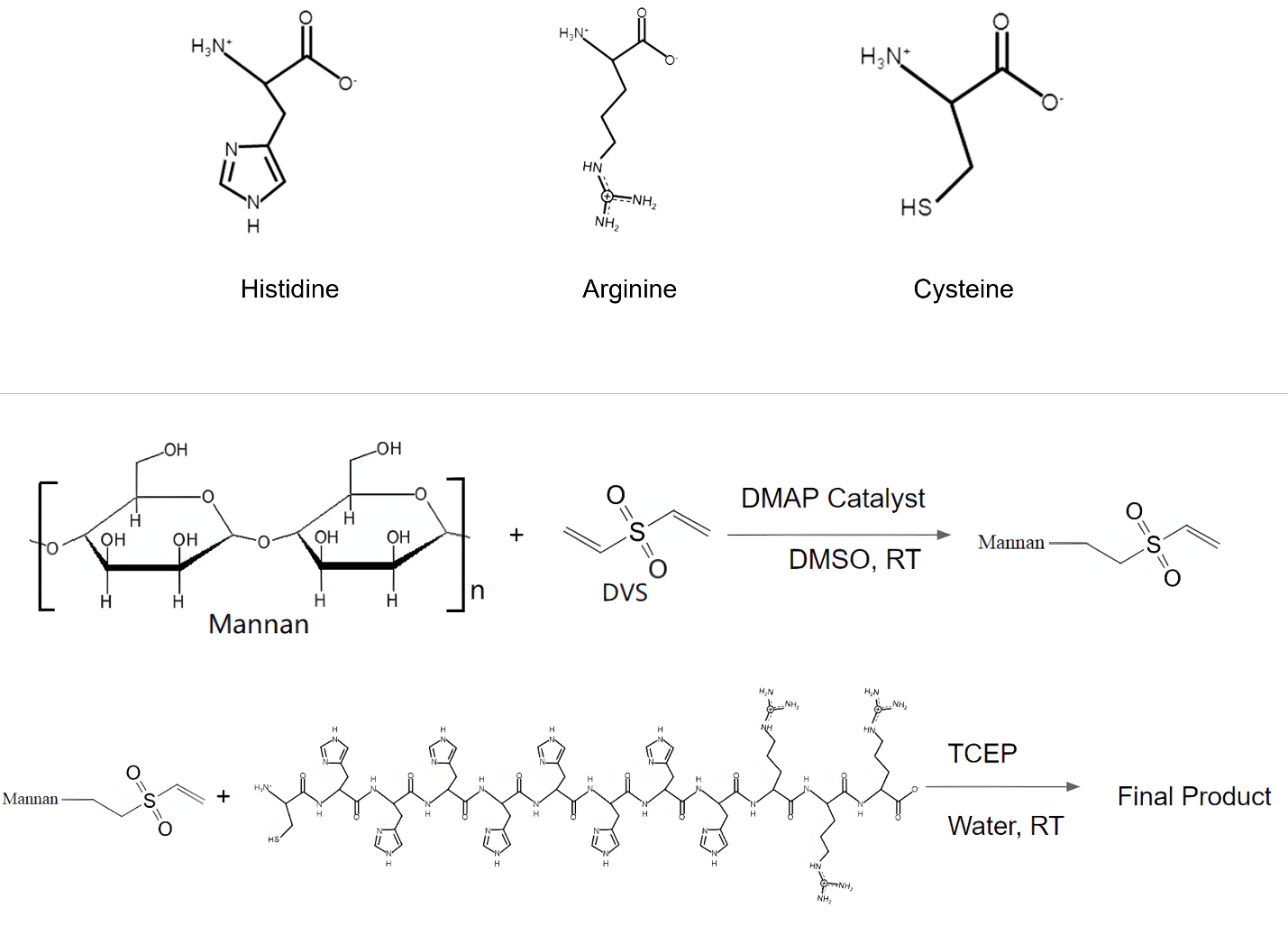


**Figure S1**. Reaction schematic illustrates the modification of mannan with the bridge link DVS, followed by conjugation with the peptide R_3_H_8_C.


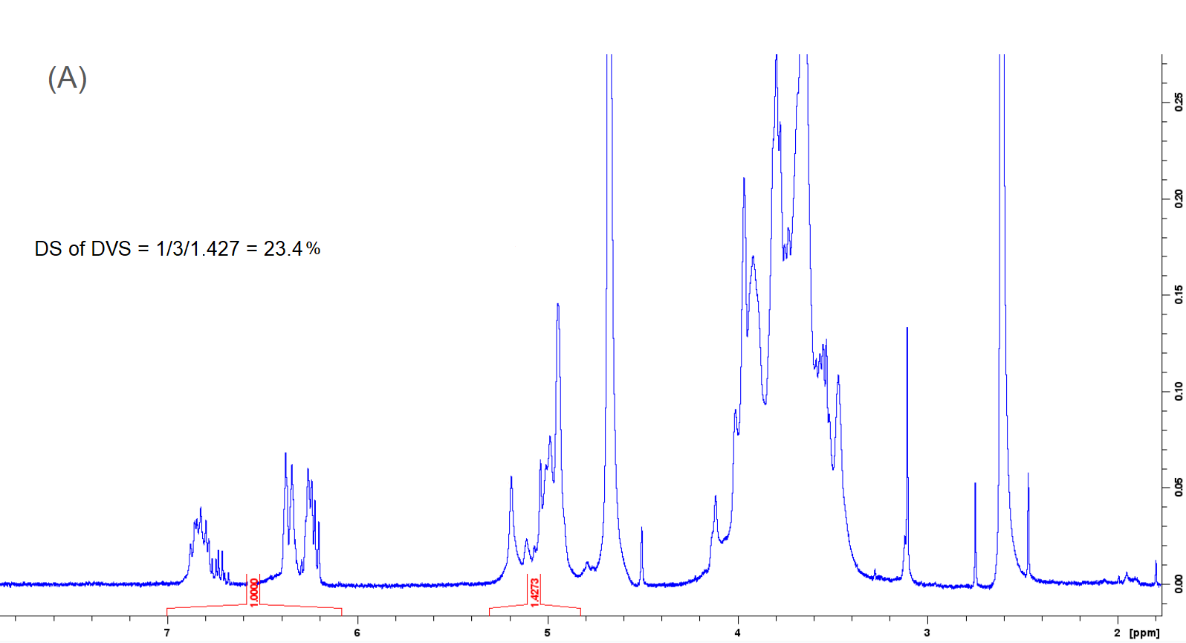


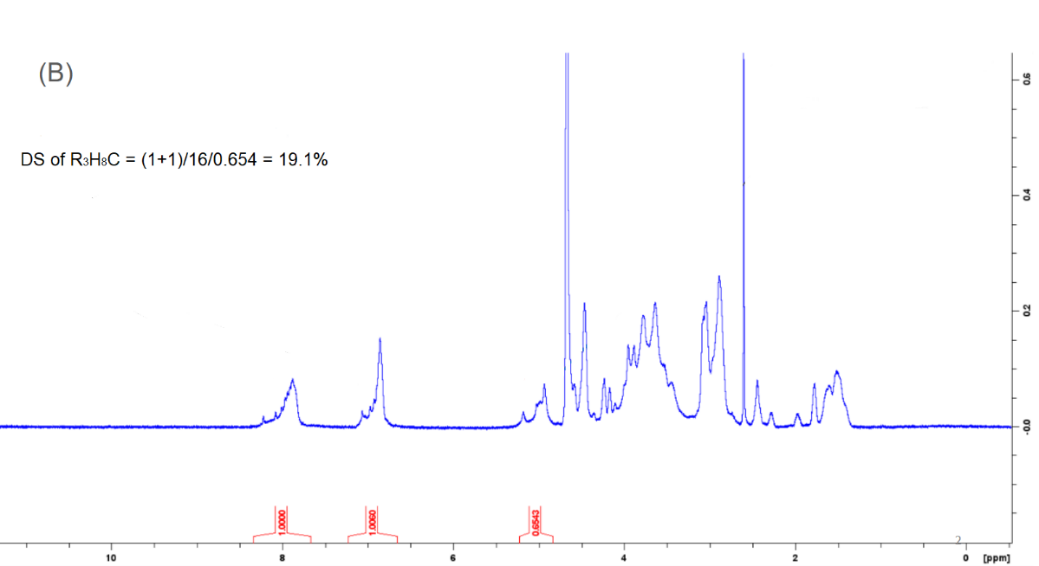


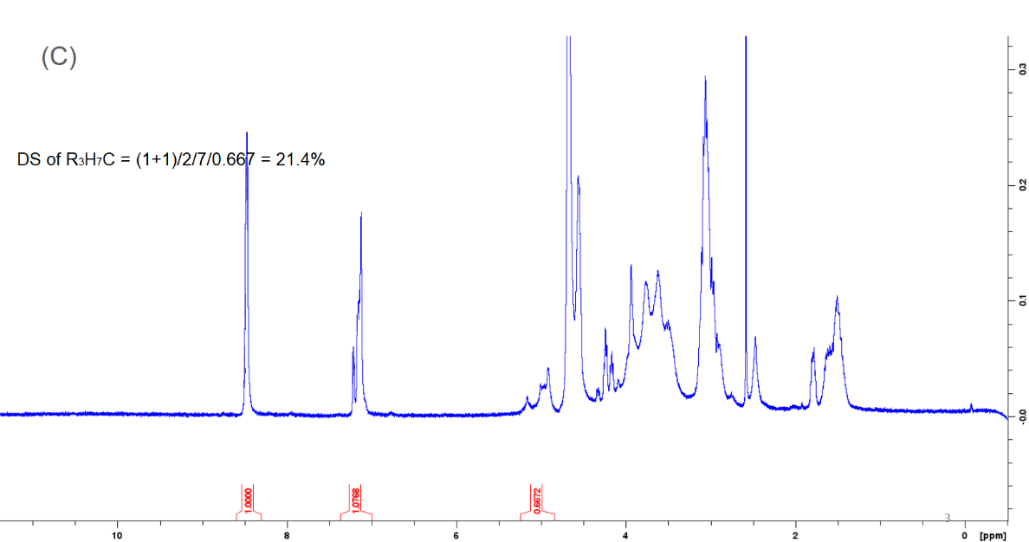


**Figure S2**. H ^1^ NMR spectra of (A) mannan-DVS, showing mannan signature peaks at 5 ppm and DVS peaks between 6-7ppm, with degree of substitute (DS) of 23.4% (B) R_3_H_8_C-mannan, showing dextran peaks at 5 ppm and peptide signature peaks around 7 and 8 ppm, with DS of 19.1% (C) R_3_H_7_C-mannan, showing dextran peaks at 5 ppm and peptide signature peaks around 7 and 8 ppm, with DS of 21.4%.


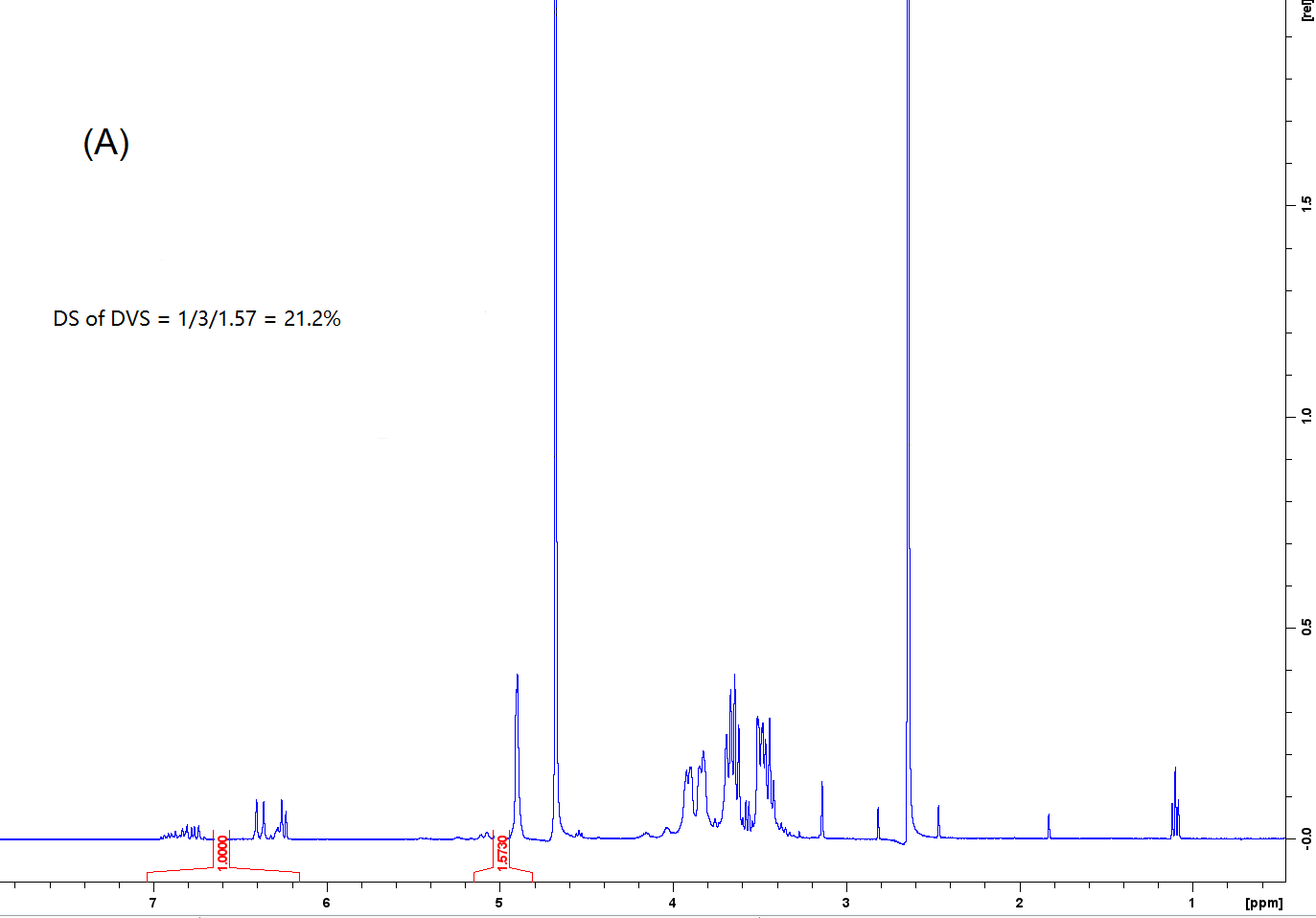


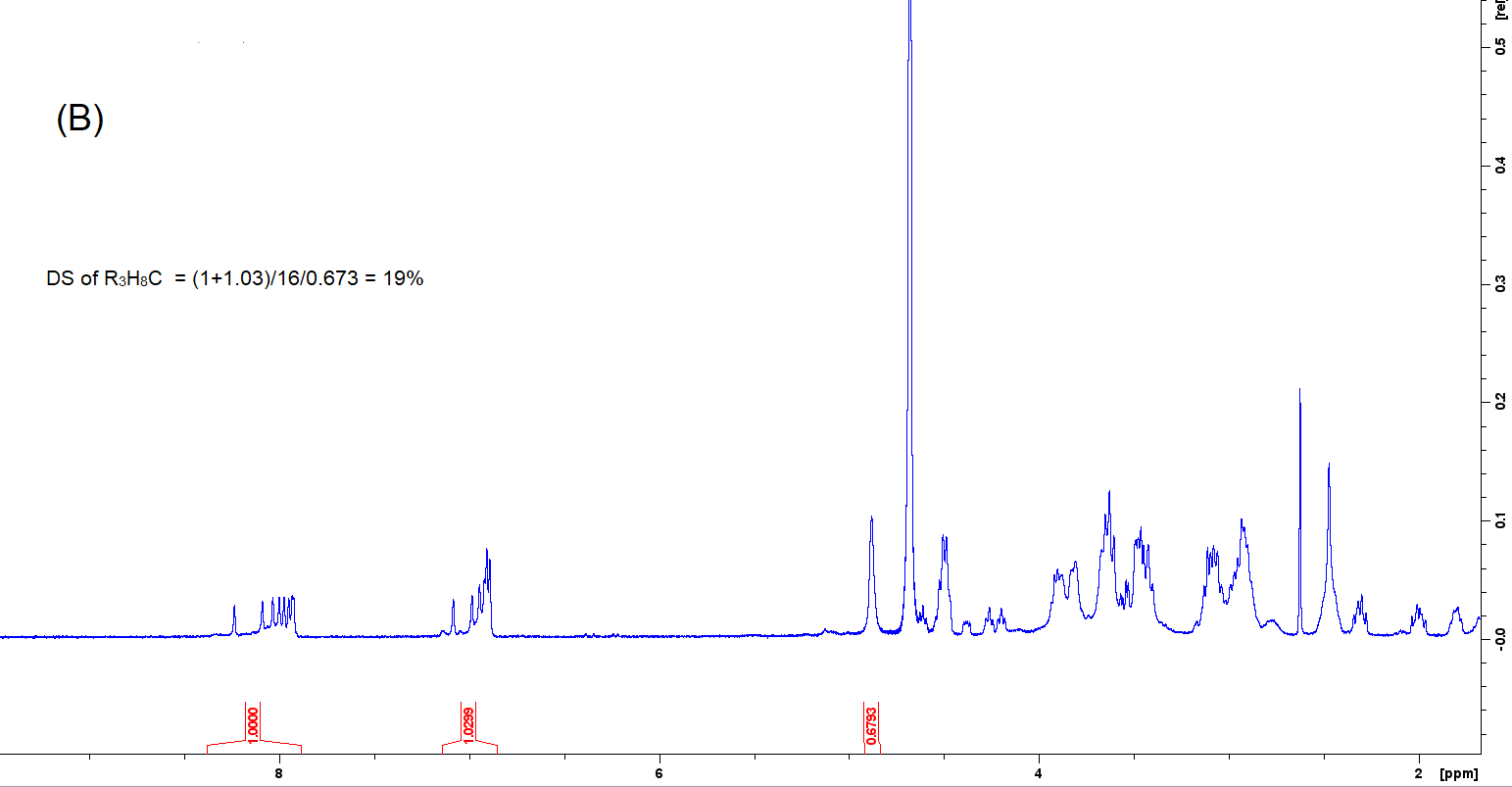


**Figure S3.** H ^1^ NMR spectra of (A) dextran-DVS, showing dextran signature peaks around 5 ppm and DVS peaks between 6-7ppm, with DS of 21.2%, (B) R_3_H_8_C-dextran, showing dextran peaks around 5 ppm and peptide signature peaks around 7 and 8 ppm, with DS of 19%.


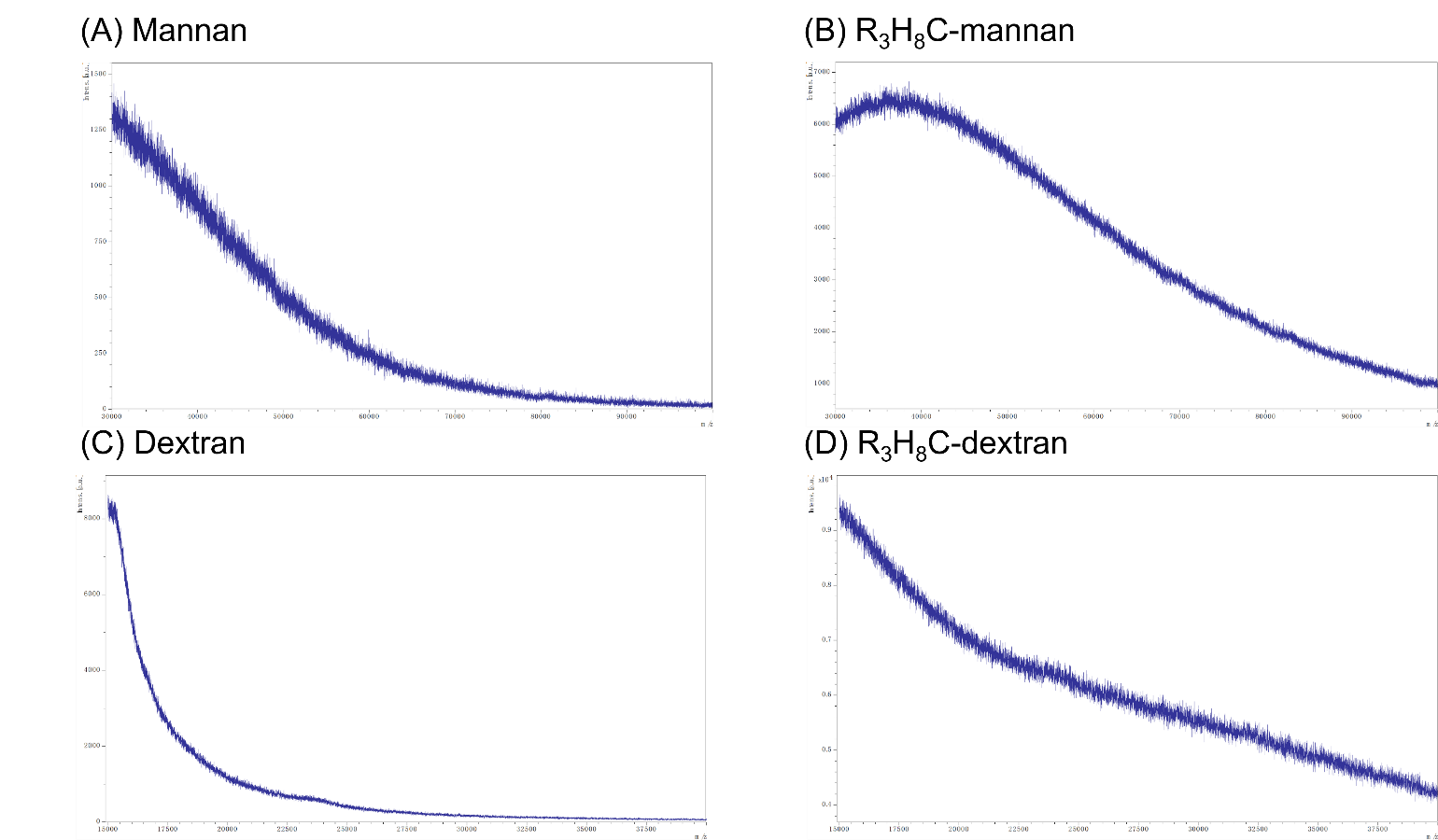


**Figure S4.** MW of mannan, dextran, and the corresponding peptide demonstrating the MW increasing after successful conjugation.

**
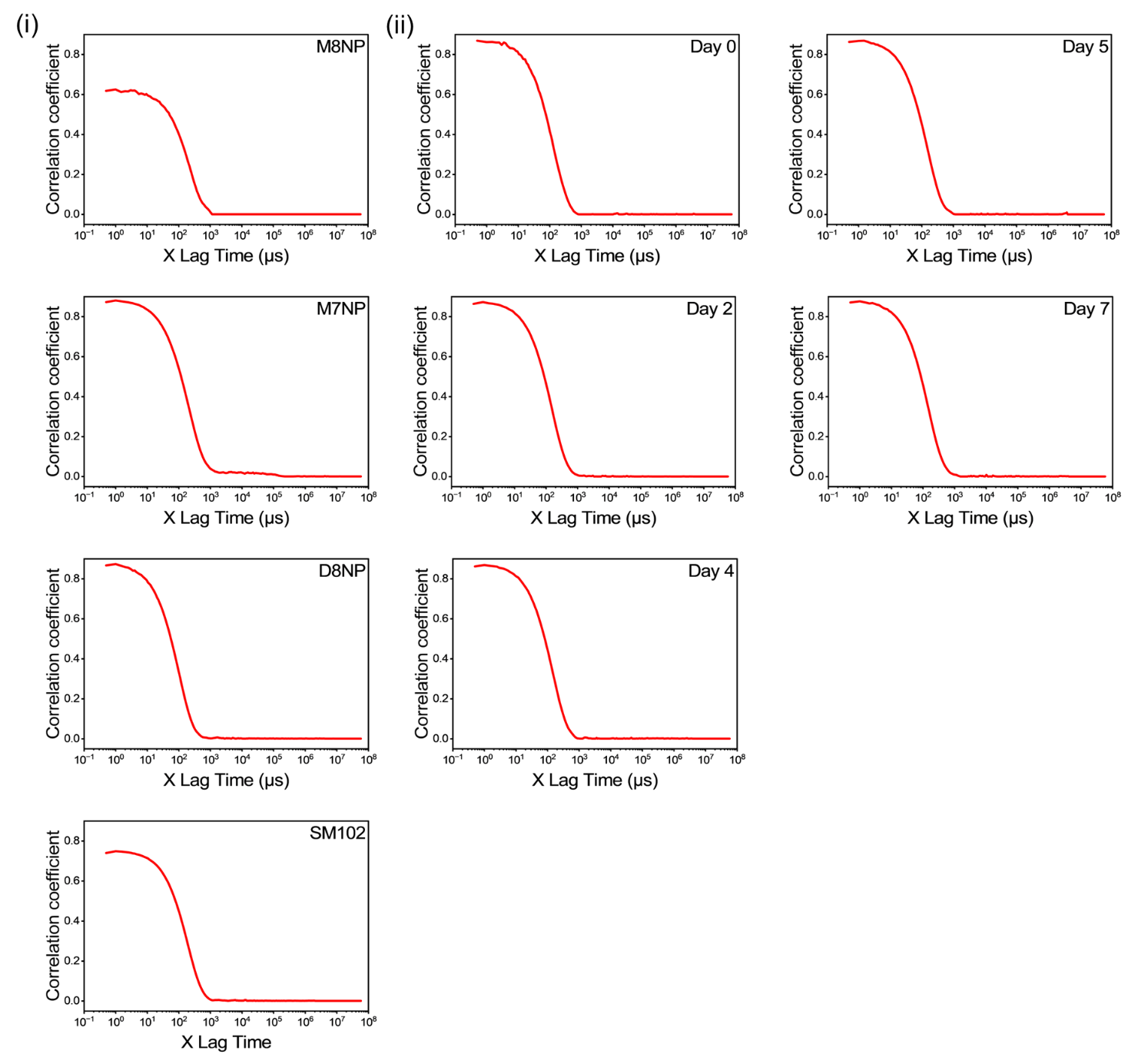
**

**Figure S5.** (i) Corresponding correlogram data in **Figure 2a(i).** (ii) Corresponding correlogram data in **Figure 2a(ii).**

**
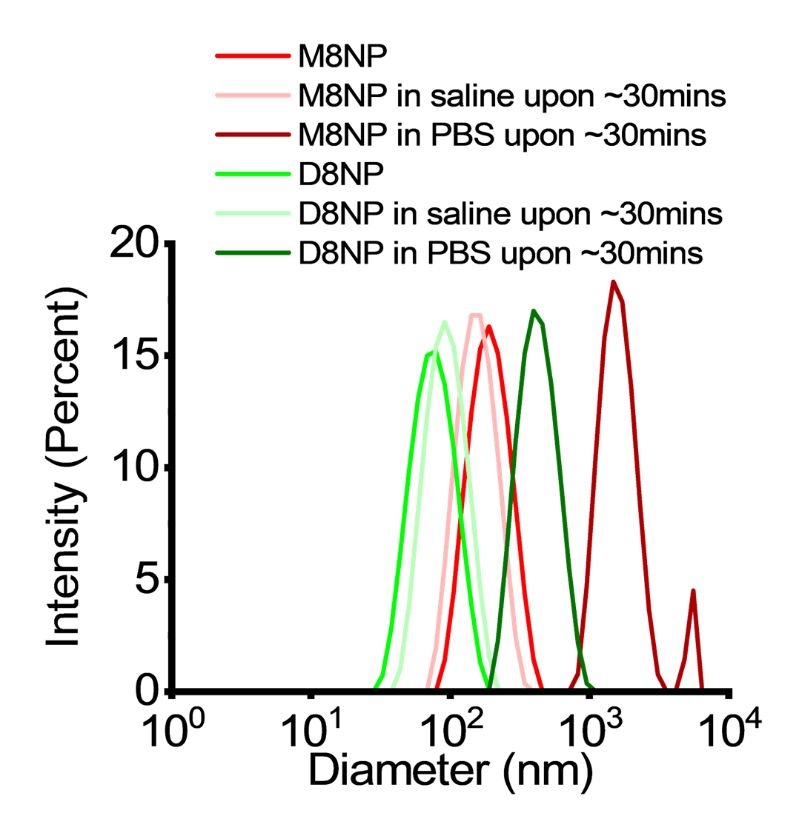
**

**Figure S6.** Stability of M8NP and D8NP in saline and PBS.

**
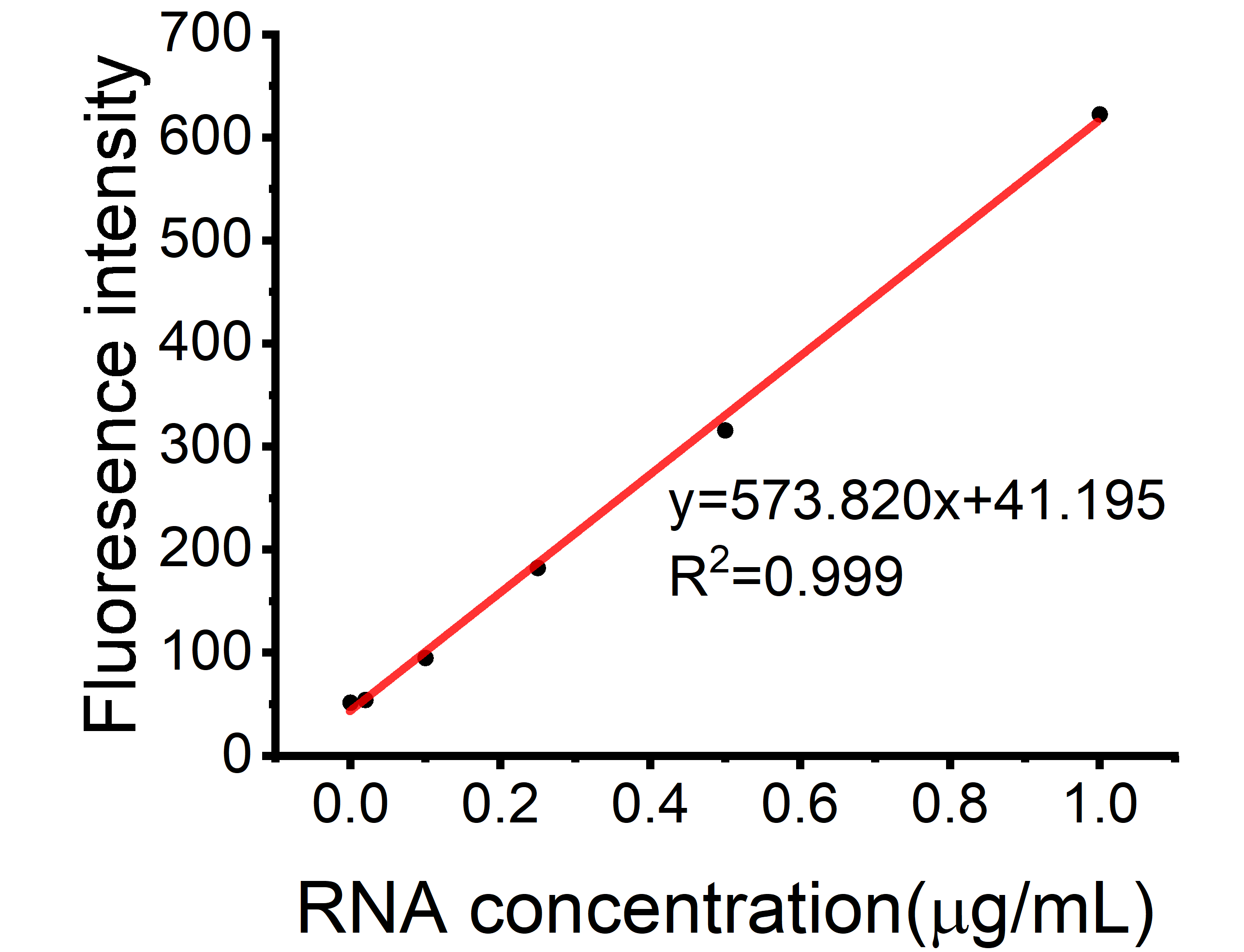
**

**Figure S7.** Standard calibration curve of the RiboGreen RNA assay with RNA concentrations ranging from 20 ng/mL to 1 µg/mL.


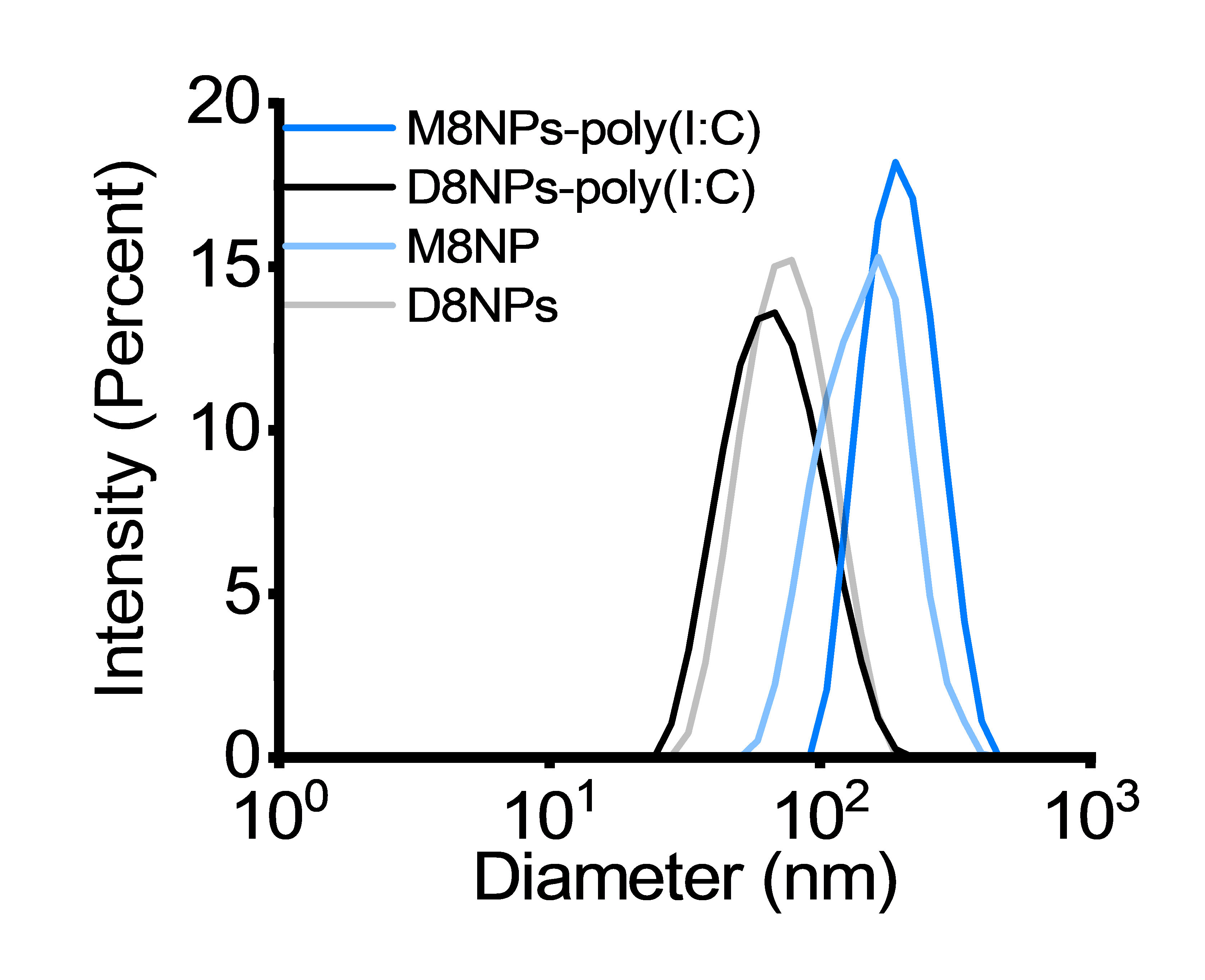


**Figure S8.** The size distribution of the poly (I:C) loaded PMNPs, compared with TGF-β loaded particles.


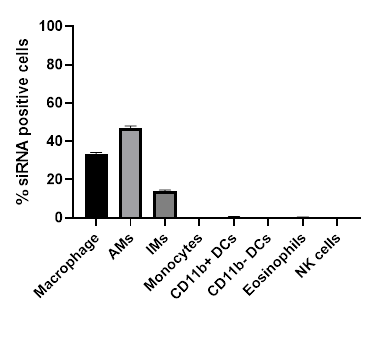


**Figure S9**. In vivo distribution of cy5 labeled siRNA following intranasal administration in fibrotic mice (day 15 post-bleomycin). The gating strategy of immune cells in flow cytometry was as follows: macrophages: CD45+Gr1-CD64+; monocytes: CD45+Ly6G-MHCII-CD64+CD11b+; dendritic cells: CD45+Ly6G-CD64-MHCII+; eosinophils: CD1b-CD45+Ly6G-CD64-MHCII-CD11b+; NK cells: CD45+Ly6G-MHCII-CD64-CD11b+. The full gating strategy is detailed in **Figure S10.**


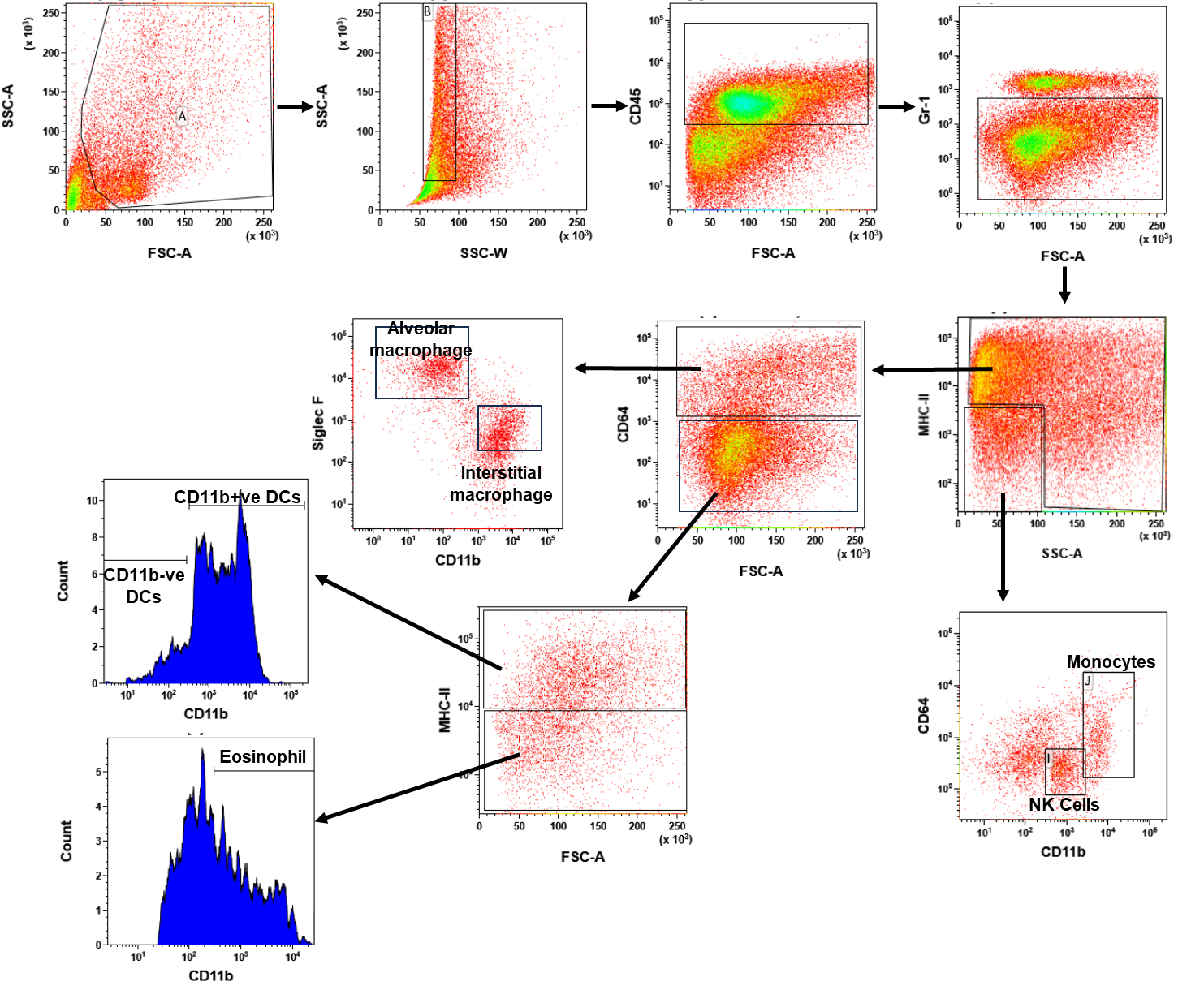


**Figure S10.** Gating strategy used to identify myeloid-cell subsets in mouse lungs for biodistribution study. Cells were isolated after enzymatic digestion of mouse lungs, and after the exclusion of doublets and debris. The gating strategy of immune cells was the following: macrophages: CD45+Gr1-CD64+; monocytes: CD45+Ly6G-MHCII-CD64+CD11b+; dendritic cells: CD45+Ly6G-CD64-MHCII+; eosinophils: CD1b-CD45+Ly6G-CD64-MHCII-CD11b+; NK cells: CD45+Ly6G-MHCII-CD64-CD11b+.


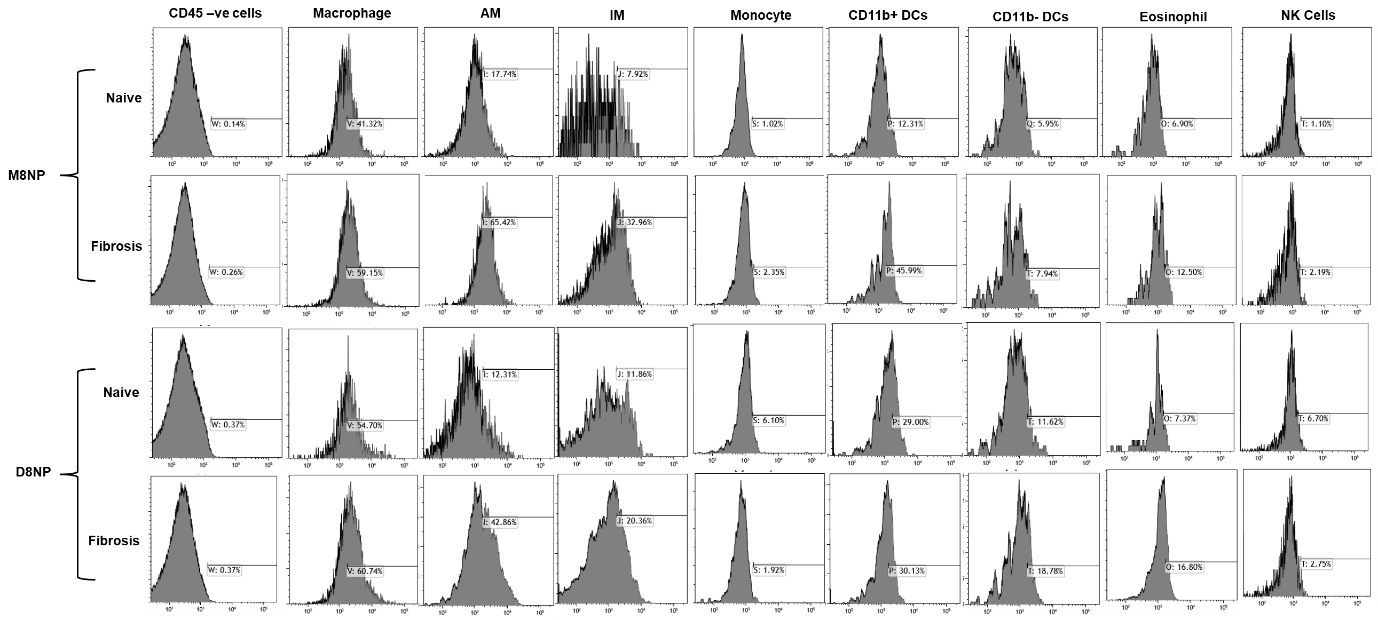


**Figure S11.** Flow cytometry histoplots showing the M8NP or D8NP positive cells. Gating strategy used to identify myeloid-cell subsets in mouse lungs for biodistribution study is described in **Figure S10**.
